# New coordinated drive mode switching strategy for distributed drive electric vehicles with energy storage system

**DOI:** 10.1038/s41598-024-56209-9

**Published:** 2024-03-18

**Authors:** Adel Oubelaid, Khoudir Kakouche, Youcef Belkhier, Nima Khosravi, Nabil Taib, Toufik Rekioua, Mohit Bajaj, Djamila Rekioua, Milkias Berhanu Tuka

**Affiliations:** 1 Faculté de Technologie, Laboratoire de Technologie Industrielle et de l’Information, Université de Bejaia, Targa ouzemour, 06000 Bejaia, Algeria; 2 Institut de Recherche de l’Ecole Navale (EA 3634, IRENav), French Naval Academy, 29240 Brest, France; 3 Centre of Research Impact and Outcome, Chitkara University Institute of Engineering and Technology, Chitkara University, Rajpura, 140401 Punjab India; 4 Department of Electrical Engineering, Graphic Era (Deemed to be University), Dehradun, 248002 India; 5 Hourani Center for Applied Scientific Research, Al-Ahliyya Amman University, Amman, Jordan; 6 Graphic Era Hill University, Dehradun, 248002 India; 7 Applied Science Research Center, Applied Science Private University, Amman, 11937 Jordan; 8 Department of Electrical Power and Control Engineering, Adama Science and Technology University, Adama, Ethiopia

**Keywords:** Hybrid electric vehicle, Drivetrain, Torque ripple, Direct torque control, Permanent magnet synchronous machines, Energy science and technology, Engineering

## Abstract

High performance and comfort are key features recommended in hybrid electric vehicle (HEV) design. In this paper, a new coordination strategy is proposed to solve the issue of undesired torque jerks and large power ripples noticed respectively during drive mode commutations and power sources switching. The proposed coordinated switching strategy uses stair-based transition function to perform drive mode commutations and power source switching’s within defined transition periods fitting the transient dynamics of power sources and traction machines. The proposed technique is applied on a battery/ supercapacitor electric vehicle whose traction is ensured by two permanent magnet synchronous machines controlled using direct torque control and linked to HEV front and rear wheels. Simulation results highlight that the proposed coordinated switching strategy has a noteworthy positive impact on enhancing HEV transient performance as DC bus fluctuations were reduced to a narrow band of 6 V and transient torque ripples were almost suppressed.

## Introduction

In 2018, Renewable Energy Sources (RES) contributed around 11% of the nation's energy consumption and 17% of power output, according to the US Energy Information Administration^1^. The ongoing and progressive shift toward non-carbon choices such as wind, solar, hydropower, biomass, and geothermal power is motivated by a number of benefits that can be described as follows^2,3^:

- RES are everlasting and never run out.
- RES are reliable and their cost is independent of political and economic instabilities.
- RES increase public health and create jobs.
- RES improve countries' economic and energy independence.
- RES are environmentally friendly.

**Figure 1:**
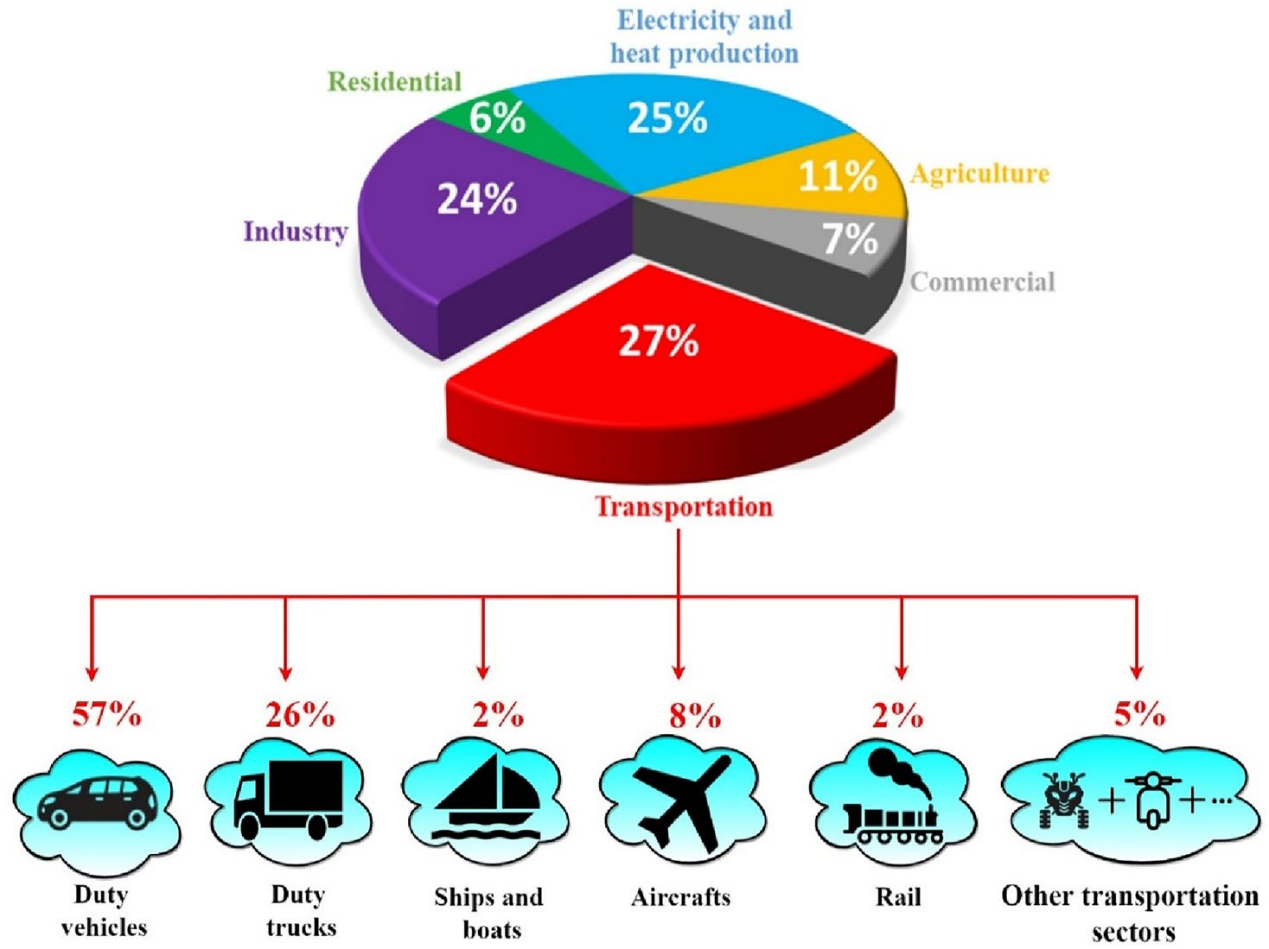
GHG emission by sector and by transportation type^1^.

Transportation market is the first polluting sector as it is illustrated in Fig. 1 which points out also that 57% of the pollution caused by the aforementioned sector is caused by duty vehicles^4,5^. This reality has prompted various countries throughout the world to investigate innovative ecologically friendly ways to replace traditional automobiles that rely on finite and dirty energy sources^6,7^. Researchers have considered electrifying the traditional traction chain, which is based on internal combustion engines, by using electric motors that emit no pollutants and are more efficient^8^. The growing interest given to electrified vehicles coupled with global world migration toward fuel economy and green technologies gave birth to several research axes^9,10^.

Fuel economy^11^, vehicular communication systems^12^, intelligent vehicles^13,14^, unmanned transportation^15^, power management algorithms^16^, Power train architecture^17^, vehicle safety^18^, grid connected electric vehicles^19^, machine control^20,21^ and power sources lifespan enhancement^22,23^ are a prominent current trend in the realm of automotive engineering^24^.

**Figure 2:**
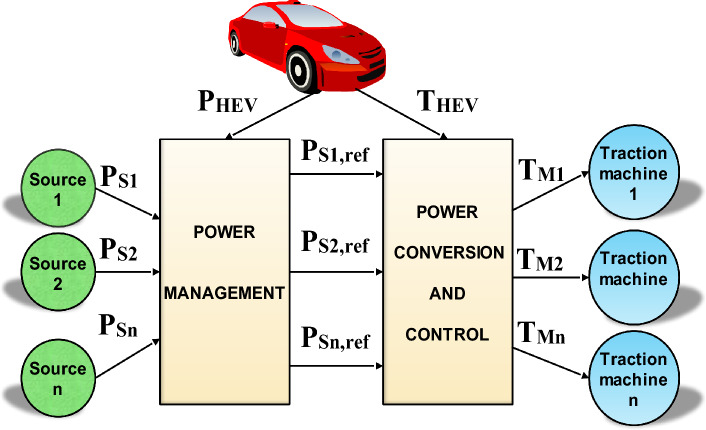
General scheme of DDHEVs^27^.

Since the introduction of hybrid electric vehicles (HEVs), the variety of powertrain architectures in vehicles has grown dramatically, which has greatly improved their performance^25,26^. As explained in^17^, the number and arrangement of electric machines on a HEV board are important scaling factors in HEV classifications. Distributing vehicle traction over machines reduces the probability of HEV failure and enhances its performances as well. Figure 2 shows a general scheme of a DDHEV with n power sources and traction motors. As it is depicted on the last-mentioned figure, power management algorithms are used to control the power flow of power sources and to condition their usage. The developed power will then be converted to torque which will be distributed conveniently over vehicle traction machines. Table 1 points out the major drivetrain structures used in DDHEV found in literature. The drivetrain architecture of type D1 uses only one traction machine connected to rear wheels which are driving the vehicle while the front wheels are being driven. The drivetrain structure D2 utilizes two traction machines connected to the rear wheels of the vehicles. The drivetrain configuration D3 employs two traction machines connected to HEV front and rear wheels. The drivetrain structure D3 enables the HEV to be driven either by front wheels or rear wheels or by front and rear wheels at the same time. One can remark that even though the same number of traction machines is used in D2 and D3, more driving modes are available in D3. This confirms that the geometrical placement of traction machines on HEV board plays and important role in determining HEV performance and characteristics. The drivetrain architecture D4 uses 4 traction machines which are connected directly to HEV wheels.

**Table 1:** Different distributed drivetrain topologies.

Drivetrain reference	Reference work	Drivetrain architecture
D1	^28,29^	
D2	^30,31^	
D3	^32,33^	
D4	^34,35^	

It is worth noticing that the type of traction machines used in the drivetrain topologies shown in Table 1 maybe of same or different nature. For instance, in^34^ two AC machines of type IM are used to drive respectively the front and rear vehicle wheels. Nevertheless, in^36,37^, the authors employed an Induction Motor (IM) and a Permanent Magnet Synchronous Motor (PMSM) to propel the rear and front vehicle wheels, respectively. Activation of the rear IM occurs exclusively when the PMSM is unable to independently manage the applied torque.

It is true that DDHEVs increase propulsion power and vehicle performance^38^. However, switching from one driving mode to another will give rise to high torque ripples and vibrations that will reduce vehicle drivability and produce undesired passenger-felt jerks. Solutions to these vibrations caused during gear shifting and rapid accelerations and decelerations were proposed in^39,40^. DDHEVs whose traction is ensured by gasoline engine and electric motors suffer from significant jerks that take place during transitions from hybrid traction mode to pure electric mode and vice versa. These jerks are due to the fact that electric motor torque and dynamics are faster than that of internal combustion engine. In^41^, a pragmatic anti-jerk approach is introduced, centering on limiting the changing rate of power-source torque to ensure a seamless mode transition process. The occurrence of jerks in HEVs is additionally attributed to miscoordination between traction power sources during drive mode transitions, as documented in^42,43^. In^44^, the authors formulated a mode transition control strategy employing state-space methodology to optimize the variation rate of vehicle acceleration, resulting in a gentle mode transition. Meanwhile, a model predictive control strategy was presented in^27,45^ with the aim of achieving a seamless mode transition. To conclude, all the reference works mentioned in this section use complex control techniques that require advanced mathematical models which makes their implementation a complex task to perform.

In this paper, a new soft transition strategy is proposed to eliminate the unsuitable transient jerks and power ripples occurring during HEV drivetrain commutations and power source switching. In addition to its simplicity of implementation, this technique enables the designer to define transition functions that compensate for the difference in dynamics and transient responses of the different power sources and traction machines within an HEV. In this study, the HEV is set to operate in rear-wheel-drive mode (RWD) as long as the load torque applied to the rear Permanent Magnet Synchronous Motor (PMSM) remains below a predefined threshold. Upon surpassing the threshold, the front PMSM is activated, transitioning the HEV into four-wheel drive mode (4WD). To optimize driving comfort and minimize jerks and vibrations, seamless transitions between RWD and 4WD are orchestrated using switching functions. These functions work to suppress transient torque ripples and ensure a consistent torque supply. Furthermore, the large transient power ripples caused by the difference in power sources dynamic responses is handled by means of adequate transition functions that ensure riding comfort and power availability. In this paper two PMSM are used to ensure HEV traction. The first machine drives the front wheels and the other is dedicated for rear wheel traction. This architecture is chosen because of the following points:

- Comfort: it enables the driver to run on RWD mode and in 4WD mode during parts of time where the torque applied on the vehicle is high.
- Security: the vehicle will not lose its stability if one machine is in failure state. Instead, it will keep running relying on one machine.

## Vehicle drivetrain and dynamics

**Figure 3:**
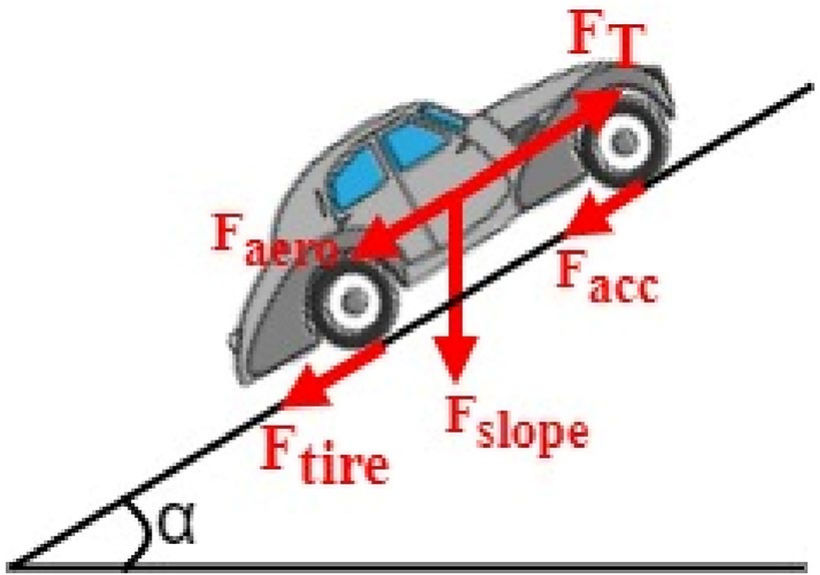
External forces acting on the HEV.

Figure 3 shows the external forces acting on the HEV as it is undergoing a given driving cycle. The dynamics of the vehicle are governed by the equation below which represents Newton’s second law:

1 $$\sum \overrightarrow{{F}_{ext}}=m\overrightarrow{a}$$

Equation (1) can be rewritten as highlighted by Eq. (2) where *F*_*T*_ is the force needed for traction and *F*_*R*_ is the net resistive force which the sum is of aerodynamic (*F*_*aero*_), slope (*F*_*slope*_), tire (*F*_*tire*_) and acceleration forces (*F*_*acc*_). More information about HEV dynamics is provided in^28^.

2 $$\overrightarrow{{F}_{T}}-\overrightarrow{{F}_{R}}=m\overrightarrow{a}$$

3 $$\overrightarrow{{F}_{R}}=\overrightarrow{{F}_{aero}}+\overrightarrow{{F}_{slope}}+\overrightarrow{{F}_{tire}}+\overrightarrow{{F}_{acc}}$$

As the vehicle is in motion, the interplay between the air in the atmosphere and the vehicle body traversing through it is termed aerodynamic drag. This force is quantified by the mathematical expression highlighted in Eq. (4). In the last-mentioned equation,* ρ* is the air density (kg/m^3^). *A* is the vehicle frontal area (m^2^), *C*_*d*_ is the aerodynamic drag coefficient (s^2^/m^2^), *V*_*wheel*_ is the vehicle longitudinal speed (m/s) and *V*_*wind*_ is the wind speed (m/s)^46,47^.

4 $${F}_{aero}=0.5\rho A{C}_{d}({V}_{wheel}+{V}_{wind}{)}^{2}$$

The slope force, stemming from road inclination, is directly proportional to the vehicle mass. It is represented by Eq. (5), where *g* is the acceleration due to gravity (m/s^2^), and α denotes the road inclination angle measured in radians.

5 $${F}_{slope}=mg\mathit{sin}(\alpha )$$

The force arising from the friction between the vehicle's tires and the ground surface is influenced by various factors, including pressure, tire deflection, ground surface (whether hard or soft), and vehicle speed. The rolling resistance force is mathematically defined by Eq. (6) as presented below, where *f*_*r*_ represents the ground rolling resistance coefficient.

6 $${F}_{tire}=mg{f}_{r}\mathit{cos}(\alpha )$$

The relationship between the ground rolling resistance coefficient and vehicle velocity is expressed by a linear equation, as depicted in Eq. (7) below:

7 $$f_{r} = 0.01\left( {1 + \frac{{V_{wheel} }}{160}} \right)$$

Acceleration force is required by the electrical vehicle to accelerate from zero to its maximum speed. This force is included in the design procedure to ensure that vehicle will overcome hills such as the one shown in Fig. 1 with its maximum speed. *F*_*acc*_ is expressed using Eq. (8) shown below where *t*_*a*_ is the time characteristic of the vehicle.

8 $$F_{acc} = m\frac{{\left( {V_{wheel} } \right)_{\max } }}{{gt_{a} }}$$

**Figure 4:**
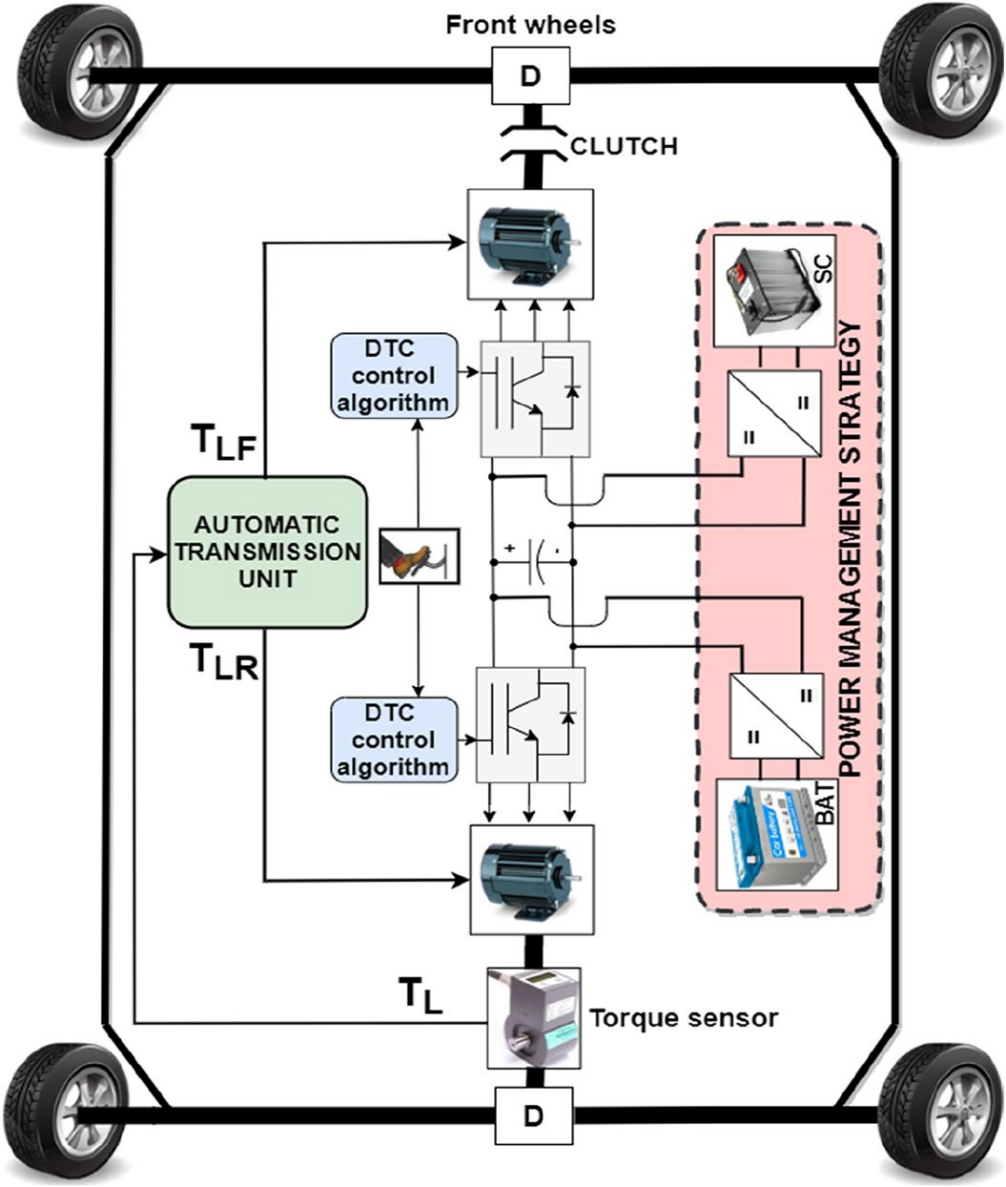
Top view of the HEV.

The powertrain architecture shown in Fig. 4 is used in this paper. One can see that it uses two permanent magnet synchronous machines (PMSM) placed between front and rear HEV wheel. This placement is expected to increase vehicle security because the HEV run on one machine if the second is in failure state. Also, the drivetrain architecture increases driving comfort since two traction modes are possible: RWD and 4WD. These machines are connected to vehicle wheels via differentials which will equally distribute the torque between wheels and ensure their rotation at different speeds as the HEV goes around corners. As long as the load torque on the rear Permanent Magnet Synchronous Motor (PMSM) shaft remains below a specified threshold value, T_TH_, the HEV operates in rear-wheel-drive (RWD) mode. In this scenario, the front PMSM shaft is disengaged from the front wheels, with the rear PMSM solely providing traction and managing the entire load torque applied to the vehicle. Upon exceeding the T_TH_ torque threshold, the front PMSM shaft automatically connects to the front wheels, transitioning the HEV into four-wheel drive (4WD). In this traction mode, both machines share the responsibility of handling half of the load torque applied to the vehicle.

**Figure 5:**
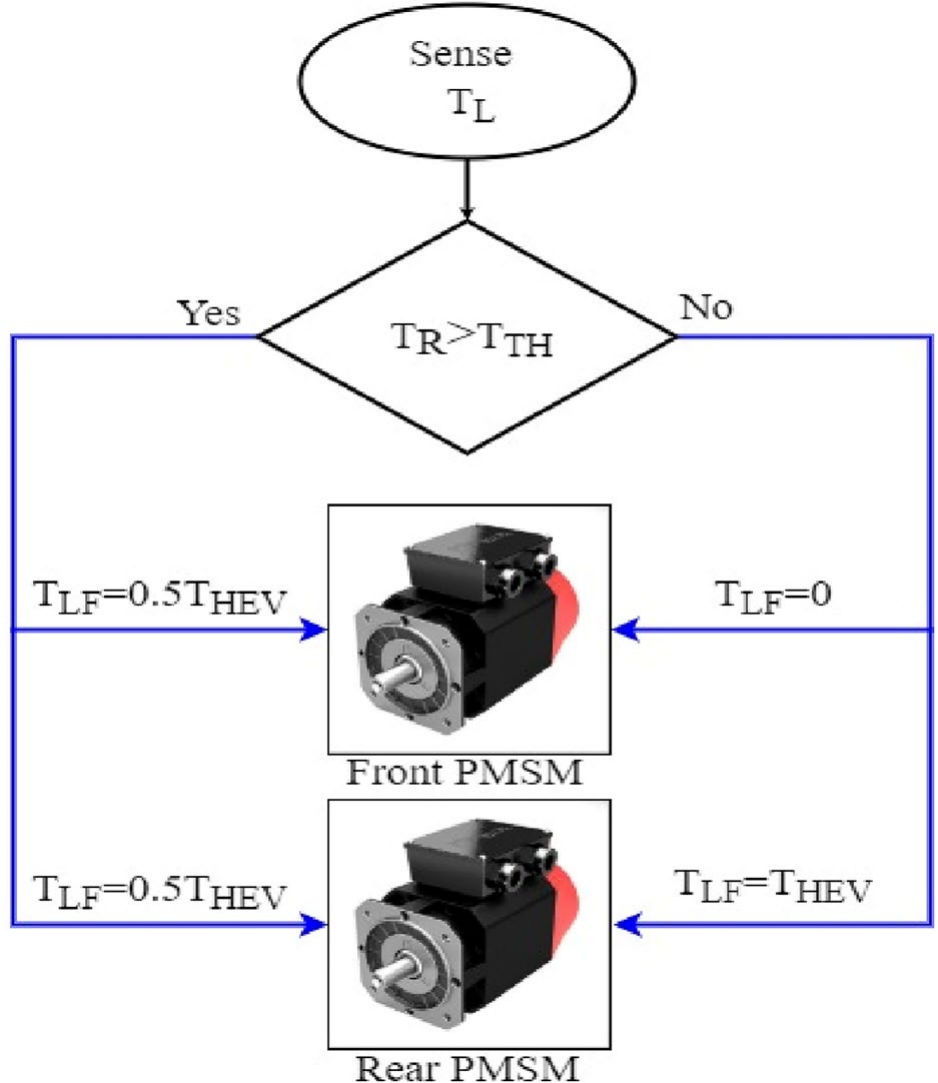
HEV traction machine operation.

The load torque applied to the HEV is expressed by Eq. (9), wherein *r* represents the wheel radius, and *G* denotes the gear ratio. Depending on the value of *T*_*L*_, either one or both traction machines will ensure vehicle traction as it is highlighted by Eq. (10) shown below where T_LF_ and T_LR_ are the values of load torque applied to the front and rear traction machines respectively. Figure 5 explains what have been said earlier about traction machine operation and illustrates how front and rear traction machines are used to ensure vehicle traction

9 $${T}_{L}=\frac{r}{G}{F}_{R}$$

10 $$T_{L} = \left\{ {\begin{array}{*{20}l} {T_{LR} } \hfill & {when\;\;\;\;\;\;\;\;\;\;\;\;\;\;\;\;\;\;\;\;\;\;\;\;0 \le T_{L} < T_{th} } \hfill \\ {T_{LF} + T_{LR} } \hfill & {when\;T_{L} \ge T_{th} \;or\;\;\;\;\;\;T_{L} \le 0} \hfill \\ \end{array} } \right.$$

## Coordinated drive mode switching

A motor shaft connection and separation from the vehicle's front wheels is implied by commutations from RWD to 4WD and vice versa. If the transition between traction modes is not handled comfortably, this may cause significant torque ripples that will impair HEV comfort and perhaps damage PMSM as well. Ripples are undesired fluctuations that are caused by several factors such as motor design, electrical noise, and abrupt load variations. In DDHEVs, drive mode commutations result in significant torque jerks which can lead to many negative consequences such as: vibration and noise, mechanical damage and reduced efficiency. In order to improve global vehicle performance and driving comfort, it is necessary to address the aforementioned disadvantages. Several techniques were proposed in literature to reduce the noteworthy bad effect of transient ripples on vehicle performance and driving comfort.

At actuator level, several control techniques were proposed to lower the impact of transient jerk on traction machines. In^45^, an advanced approach is introduced to mitigate torque ripples and enhance the anti-disturbance capability of permanent magnet synchronous motors. This is achieved through a robust iterative learning control scheme implemented by adaptive sliding mode control. In^48^, model predictive control is coupled with fuzzy logic to minimize the torque ripples of the permanent magnet motor driving the electric vehicle. Authors in^49^ have used neural networks for the minimization of torque ripples in non-sinusoidal synchronous reluctance motors. In^50^ and^51^, authors have used, respectively, direct torque control-based space vector modulation and fuzzy logic control to lower the impact of torque oscillation on the traction motor. To summarize, even though all the previously mentioned research works contribute in reducing torque ripples, they still suffer from many drawbacks. For instance, at software level, all the previously mentioned control techniques suffer from time complexity as they are all based on complex mathematical models. The drawbacks of each of the previously mentioned techniques dedicated for ripple minimization are summarized in Table 2 shown below.

**Table 2:** Different torque minimization techniques at actuator level.

Technique	Drawbacks	References
Direct torque control-based space vector modulation	High switching frequency Slow speed response during low speed and sudden change in b torque command Time complexity	^50^
Model predictive control	Computational complexity High implementation cost Dependence to cost function and	^48^
Sliding mode control	High-frequency oscillation around the sliding surface (chattering) Limited application domain: used only for slow or moderate dynamic systems Lack of smoothness: SMC is a discontinuous control technique	^45^
Fuzzy logic control	Heavy time complexity for large scale systems Lack of interpretability: no general approach for rule design Complete dependance on human knowledge and expertise	^51^
Artificial neural networks	Overfitting Computational complexity Need for large amounts of data	^49^

At power sources level, many research papers have discussed the enhancement of vehicle transient performance and the protection of power sources during sudden load variations. For instance, in^52^, authors have limited reference power slope to prevent power sources from drawing large and abrupt currents that may cause their damage. In^53^, authors have used first order transfer functions as filters to deliver the type of reference power corresponding to the transient dynamics of each power source. The last-mentioned strategies protect power sources from many undesired phenomena such as overheating and oxygen starvation but they still don’t compensate for the difference in transient dynamics between power sources. Unlike the previously control techniques at motor and power sources level, the proposed coordinated switching strategy enables the following:

- At motor level, the new coordinated switching strategy addresses the unwanted transient torque ripples occurring during drivetrain commutations and this enhances driving comfort and vehicle performance.
- At power sources level, the proposed coordinated switching strategy compensate the transient dynamics between slow and fast power sources and controls the switching period duration through transition functions fitting the transient dynamics of power sources. This eliminates the transient power peaks noticed during power sources switchings.

In contrast to the previously mentioned control techniques at both motor and power sources level, the proposed coordinated switching strategy is simple and doesn’t require complex mathematical model and does not require prior knowledge of system parameters. This makes the proposed coordinated control strategy presented in this paper a very interesting for use. In this work, the unwanted torque ripples that occur during RWD/4WD and vice versa will be minimized using a proposed commutation algorithm which will ensure soft transition between the different traction modes.

As stated in the introduction, drive mode switching in DDHEV have been widely investigated in recent years because they contribute in enhancing vehicle comfort and drivability by eliminating the undesired passenger-felt jerks during mode transition. To evaluate the detrimental impact of torque ripples on vehicle performance, a coordination switching strategy is proposed in this section. At any given instant, the torque generated by the HEV, as depicted in Fig. 4, is articulated through Eq. (11) provided below. Here, *c*_*m1*_ and *c*_*m2*_ represent the torque contribution factors of PMSM1 and PMSM2, respectively, connected to the front and rear vehicle wheels. Each factor signifies the percentage of the total load torque handled by the respective machine. When HEV traction relies solely on PMSM1, *c*_*m1*_ equals 100%, and *c*_*m2*_ is zero. Upon the HEV transitioning to dual traction mode, both *c*_*m1*_ and *c*_*m2*_ are set to 50%.

11 $$\left\{\begin{array}{l}{T}_{HEV}\hspace{0.33em}={c}_{m1}{T}_{m1}+{c}_{m2}{T}_{m2}\\ {\sum }_{i=1}^{2}{c}_{mi}=1\end{array}\right.$$

By taking the time derivative of Eq. (11) we get the mathematical expression of instantaneous HEV torque ripples as indicated by Eq. (12). The numerical approximation for each right-hand side term of Eq. (12) is outlined in Eq. (13). Here, *T*_*s*_ in Eq. (13) represents the calculation step. The derivatives of *T*_*m1*_ and *T*_*m2*_ are set to zero because the torque developed by the two traction machines between *t* and t + *T*_*s*_ remains the same. With these approximations, Eq. (12) can be expressed as indicated in Eq. (14).

12 $$\frac{d{T}_{HEV}}{dt}=\left[{T}_{m1}\frac{d{c}_{m1}}{dt}+{T}_{m2}\frac{d{c}_{m2}}{dt}\right]+\left[{c}_{m1}\frac{d{T}_{m1}}{dt}+{c}_{m2}\frac{d{T}_{m2}}{dt}\right]$$

13 $$\left\{\begin{array}{c}\frac{d{T}_{m1}}{dt}\approx \left[\frac{{T}_{m1}\left(t+{T}_{s}\right)-{T}_{m1}\left(t\right)}{{T}_{s}}\right]\approx 0\\ \frac{d{T}_{m2}}{dt}\approx \left[\frac{{T}_{m2}\left(t+{T}_{s}\right)-{T}_{m2}\left(t\right)}{{T}_{s}}\right]\approx 0\\ \frac{d{c}_{m1}}{dt}\approx \left[\frac{{c}_{m1}\left(t+{T}_{s}\right)-{c}_{m1}\left(t\right)}{{T}_{s}}\right]\approx \frac{\Delta {c}_{m1}}{{T}_{s}}\\ \frac{d{c}_{m2}}{dt}\approx \left[\frac{{T}_{m2}\left(t+{T}_{s}\right)-{T}_{m2}\left(t\right)}{{T}_{s}}\right]\approx \frac{\Delta {c}_{m2}}{{T}_{s}}\end{array}\right.$$

14 $$\frac{d{T}_{HEV}}{dt}\approx {T}_{m1}\left(\frac{\Delta {c}_{m1}}{{T}_{s}}\right)+{T}_{m2}\left(\frac{\Delta {c}_{m2}}{{T}_{s}}\right)$$

Due to *T*_*s*_ being in the order of 10^–6^, the two ratios in the right-hand side of Eq. (14) lead to substantial transient torque ripples, compromising HEV driving comfort during drive mode switching and potentially diminishing the lifespan of the Permanent Magnet Synchronous Motors (PMSMs) and overall driving comfort. To mitigate these drawbacks, this paper suggests a coordinated switching strategy that streamlines drive mode transitions and harmonizes the torque output from the two traction machines. The old and the new torque contribution factors of PMSM_1_ denoted respectively by ${c}_{m1}^{old}$ and ${c}_{m1}^{new}$ are acquired and stored as given in Eq. (15) where *t*_*trig*_ is the instant at which* T*_*L*_ gets beyond or blow *T*_*TH*_. The same is task is performed for PMSM2 as it is indicated in Eq. (16) shown below.

15 $$\left\{\begin{array}{c}{c}_{m1}^{old}={c}_{m1}\left({t}_{trig}-{T}_{s}\right)\\ {c}_{m1}^{new}={c}_{m1}\left({t}_{trig}\right)\end{array}\right.$$

16 $$\left\{\begin{array}{c}{c}_{m2}^{old}={c}_{m2}\left({t}_{trig}-{T}_{s}\right)\\ {c}_{m2}^{new}={c}_{m2}\left({t}_{trig}\right)\end{array}\right.$$

The old and new reference torque to be applied to the front and rear traction machines are respectively calculated using Eqs. (17) and (18) respectively as shown below:

17 $$\left\{\begin{array}{c}{T}_{m1}^{old}={c}_{m1}^{old}{T}_{L}\left({t}_{trig}-{T}_{s}\right)\\ {T}_{m1}^{new}={c}_{m1}^{new}{T}_{L}\left({t}_{trig}\right)\end{array}\right.$$

18 $$\left\{\begin{array}{c}{T}_{m2}^{old}={c}_{m2}^{old}{T}_{L}\left({t}_{trig}-{T}_{s}\right)\\ {T}_{m2}^{new}={c}_{m2}^{new}{T}_{L}\left({t}_{trig}\right)\end{array}\right.$$

**Figure 6:**
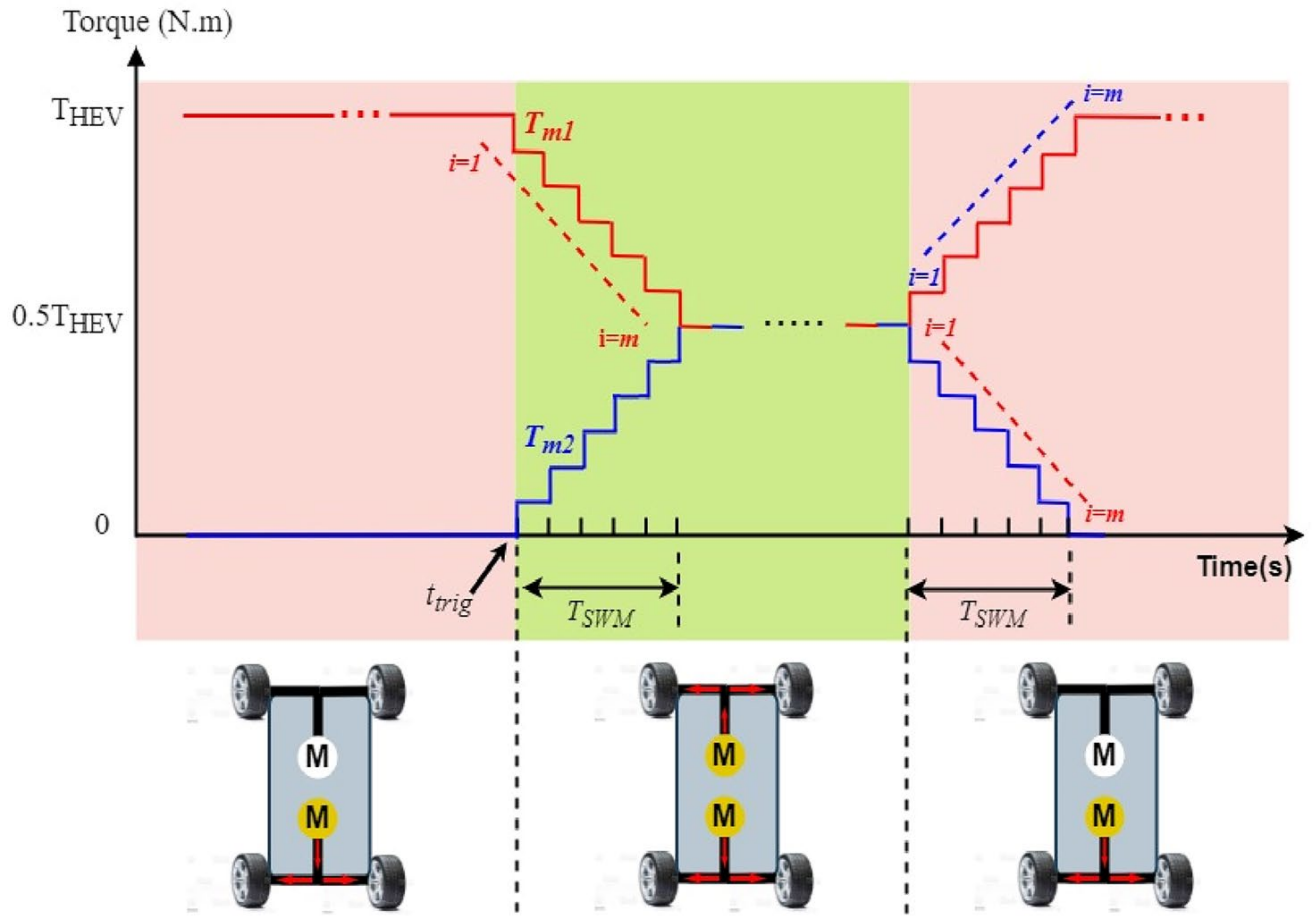
HEV stair-based coordinated mode switching.

Using the stair based transition functions highlighted by Eqs. (19) and (20), torque transition from ${T}_{m1}^{old}$ to ${T}_{m1}^{new}$ for front PMSM and torque commutation from ${T}_{m2}^{old}$ to ${T}_{m2}^{new}$ for rear PMSM are splitted into *m* allowable sub transitions performed within a transition period *T*_*SWM*_ defined by Eq. (21) which determines the relation between the sub transient period δ_FC_ and the total transition period T_SWM_. Using the two stair-based transition function shown below, the front and rear traction machines will receive an increasing/decreasing torque references each δ second during [*t*_*trig*_*; t*_*trig*_ + *T*_*SWM*_] till it reaches its new reference power after T_SWM_ seconds. It is worth noticing that *i* in Eq. (19)–(20) is an integer ranging from 1 to *m.* Figure 6 shown below highlights how the HEV toggles between RWD and 4WD modes using the proposed stair-based coordinated switching strategy. From the aforementioned figure, one can notice that when front and rear traction machines are both ON each of them handles half of the load torque applied on the vehicle. Whereas when T_HEV_ is below T_TH_ the vehicle runs only using the rear traction machine. Figure 6 highlights clearly that commutations from RWD to 4WD and vice versa are performed within T_SWM_ seconds.

19 $${T}_{m1}^{ref}\left({t}_{trig}+i\upxi \right)={T}_{m1}^{old}+i\left(\frac{{T}_{m1}^{new}-{T}_{m1}^{old}}{m}\right)$$

20 $${T}_{m2}^{ref}\left({t}_{trig}+i\upxi \right)={T}_{m2}^{old}+i\left(\frac{{T}_{m2}^{new}-{T}_{m2}^{old}}{m}\right)$$

21 $${T}_{SWM}=m\upxi$$

**Figure 7:**
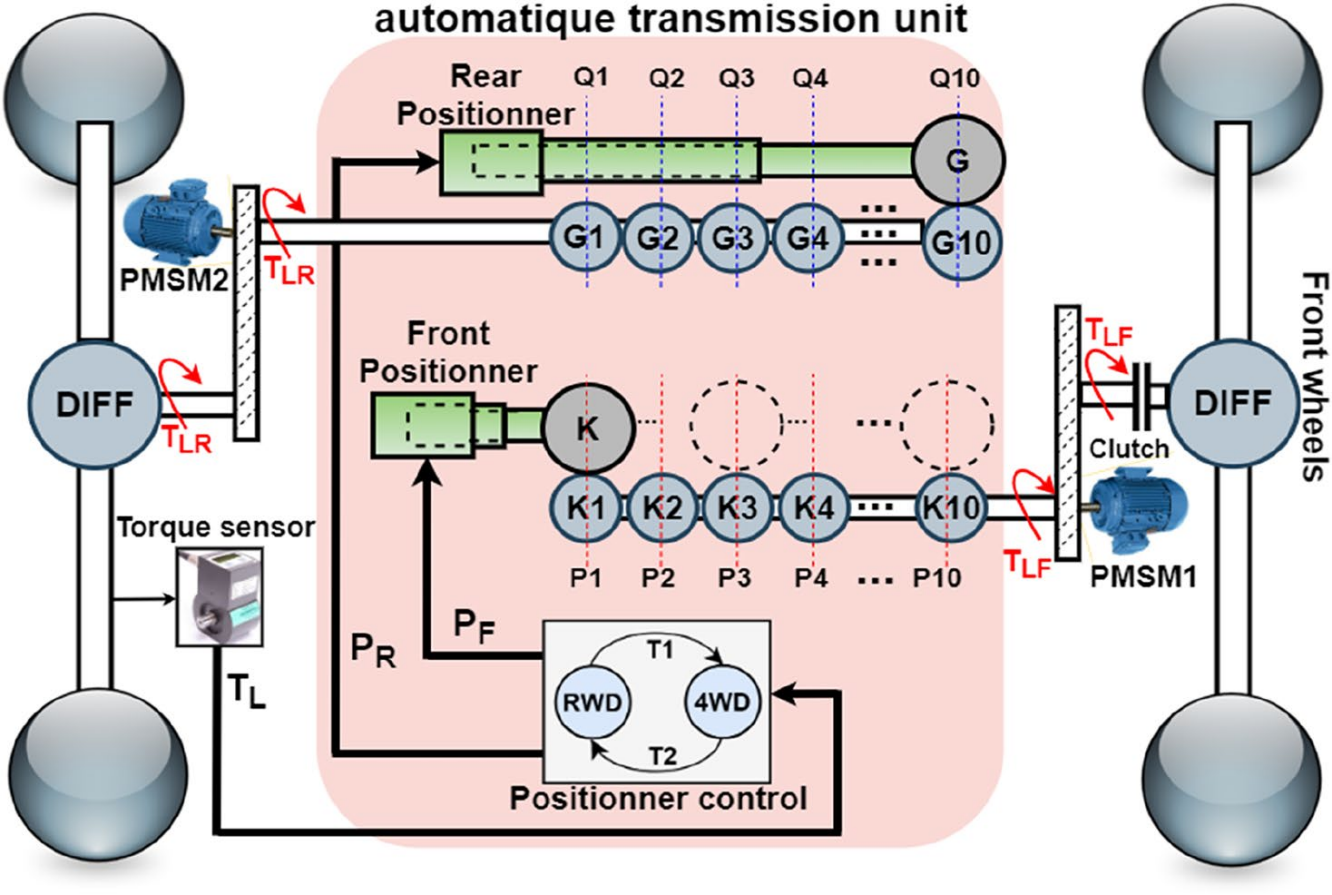
Automatic transmission unit internal structure.

Figure 7 shows one possible mechanical implementation of the proposed coordinated drive mode switching using stair-based transition function. It is worth noticing that Fig. 7 is no more than the internal structure of the automatic transmission block already presented in Fig. 4. From Fig. 7 shown below, one can see that the torque sensor measures the load torque applied on rear wheels and provides that value to the positioner control block which will output two control signals P_R_ and P_F_. These two last mentioned signals will control the position of gears G and K fixed on the front and rear positioner respectively as pointed out in Fig. 7. The ratio between the sliding gear fixed on the front positioner and the gears coupled with the front motor shaft at each position Pi is given by Eq. (22) where n is the number of transition gears coupled to front PMSM shaft which are fixed to 10 in this paper. β is a reduction coefficient between each two successive gears which is set to 5%. Hence, the torque will be equally shared between front and rear PMSMs after 10 gear stages. The ratio between the sliding gear fixed on the rear positioner and the gears coupled with the rear motor shaft at each position *K*_i_ is given by Eq. (23).

22 $$\frac{{K}_{n}}{K}=\beta n$$

23 $$\frac{{G}_{n}}{G}=1-\frac{{K}_{n}}{K}$$

As the load torque exceeds the threshold value, the clutch will be closed and front PMSM shaft will be connected to front wheels. In this case, the torque of the front PMSM, T_LF_, will increase progressively from zero to half the load torque applied on the HEV according to Eq. (24) where: *t*_*tr*_ is the instant at which the 4WD mode is triggered. ξ is the time period elapsed during the transition between two successive gear positions. It is defined using Eq. (25) in which *T*_*SWM*_ is the switching mode period. The control signal *P*_*F*_ will move the front positioner from position P_1_ to P_10_. At each time instant, the placement of the front positioner is described using Eq. (26):

24 $${T}_{LF}\left[{t}_{tr}+\left(n-1\right)\xi ,{t}_{tr}+n\xi \right]=\frac{{K}_{n}}{K}{T}_{L}\left[{t}_{tr}+\left(n-1\right)\xi \right]$$

25 $$\xi =\frac{{T}_{SWM}}{n}$$

26 $${P}_{F}\left[{t}_{tr}+\left(n-1\right)\xi ,{t}_{tr}+n\xi \right]={P}_{n}$$

Simultaneously during drivetrain transition detection, the control signal P_R_ will move the rear positioner connected to PMSM_2_ from position Q_1_ to Q_10_. This will decrease by half the torque applied on the rear machine and reduce the stress on it. This torque decay is governed by Eq. (27) shown below. The location of the rear positioner P_R_ at each $\xi$ is given by Eq. (28) shown below:

27 $${T}_{LR}\left[{t}_{tr}+\left(n-1\right)\xi ,{t}_{tr}+n\xi \right]=\frac{{G}_{n}}{G}{T}_{L}\left[{t}_{tr}+\left(n-1\right)\xi \right]$$

28 $${P}_{R}\left[{t}_{tr}+\left(n-1\right)\xi ,{t}_{tr}+n\xi \right]={Q}_{n}$$

As the load torque gets below the threshold value, the HEV will toggle from 4WD to RWD mode. The torque applied on the front machine shaft will be progressively decreased by moving back at each ξ the front positioner from position P_10_ to position P_1_ till opening the clutch. During this transition, front machine torque will be governed using Eq. (29) shown below. The location of the front positioner P_F_ at each time instant ξ is given using Eq. (30)

29 $${T}_{LF}\left[{t}_{tr}+\left(n-1\right)\xi ,{t}_{tr}+n\xi \right]=\frac{{K}_{10-n}}{K}{T}_{L}\left[{t}_{tr}+\left(n-1\right)\xi \right]$$

30 $${P}_{F}\left[{t}_{tr}+\left(n-1\right)\xi ,{t}_{tr}+n\xi \right]={P}_{10-n}$$

During 4WD to RWD transition, the traction machine connected to rear wheels will handle all the HEV load torque applied on the HEV. During this drive mode transition, the torque applied on the rear machine will increase progressively according to Eq. (31) and the rear positioner will move from its limit position Q_10_ to its initial position Q_1_ following Eq. (32) shown below:

31 $${T}_{LR}\left[{t}_{tr}+\left(n-1\right)\xi ,{t}_{tr}+n\xi \right]=\frac{{G}_{10-n}}{G}{T}_{L}\left[{t}_{tr}+\left(n-1\right)\xi \right]$$

32 $${P}_{R}\left[{t}_{tr}+\left(n-1\right)\xi ,{t}_{tr}+n\xi \right]={Q}_{10-n}$$

The proposed coordinated switching strategy is also applied at power sources level to compensate the difference in dynamics between the used energy sources used to power the vehicle in this work which are battery and supercapacitor. Following the same approach as above and by using the stair based transition function given by Eqs. (33)–(34), fuel cell and battery variations from ${P}_{bat}^{old}$ to ${P}_{bat}^{new}$ and from ${P}_{SC}^{old}$ to ${P}_{SC}^{new}$ will be splitted into *m* allowable sub transitions performed within a transition period of *T*_*SWS*_ seconds as indicated by Eq. (35). This last equation which defines the relation between the sub transient period δ_bat_ and the total transition period T_SWS_. *j* in Eqs. (33)–(34) is an integer ranging from 1 to *m*. Using the stair-based transition function, both battery and supercapacitor will receive an increasing/decreasing power references each δ_FC_ second during [*t*_*trig*_*; t*_*trig*_ + *T*_*SWS*_] till it reaches its new reference power after T_FC_ seconds.

33 $${P}_{bat}^{ref}\left({t}_{trig}+j{\delta }_{bat}\right)={P}_{bat}^{old}+j\left(\frac{{P}_{bat}^{new}-{P}_{old}^{new}}{m}\right)$$

34 $${P}_{SC}^{ref}\left({t}_{trig}+j{\delta }_{SC}\right)={P}_{SC}^{old}+j\left(\frac{{P}_{SC}^{new}-{P}_{SC}^{new}}{m}\right)$$

35 $$\left\{\begin{array}{l}{\delta }_{SC}={\delta }_{bat}\\ {T}_{SWS}=m{\delta }_{SC}\end{array}\right.$$

## PMSM modeling and control

Permanent magnet synchronous machines are extensively used in traction application such as hybrid electric vehicles because of their robustness, small size, wide operational speed range, large overload capacity and high torque to mass ratio. This section deals with the modeling and the control of permanent magnet synchronous machine which ensures electric vehicle traction. Stator flux and voltages on the direct and quadrature axes are respectively given by Eqs. (36) and (37) shown below:

36 $$\left\{\begin{array}{l}{\lambda }_{d}={L}_{d}{i}_{d}+\phi \\ {\lambda }_{q}={L}_{q}{i}_{q}\end{array}\right.$$

37 $$\left\{\begin{array}{l}{V}_{d}={r}_{s}{i}_{d}+\frac{d{\lambda }_{d}}{dt}-{w}_{e}{\lambda }_{q}\\ {V}_{q}={r}_{s}{i}_{q}+\frac{d{\lambda }_{q}}{dt}-{w}_{e}{\lambda }_{d}\end{array}\right.$$

where *r*_*s*_ is the stator resistance,* i*_*d*_ is the d-axis stator current, *i*_*q*_ is the q-axis stator current, *λ*_*d*_ is the d-axis flux linkage, *λ*_*q*_ is the q-axis stator flux linkage. *L*_*d*_ is the d-axis inductance, *L*_*q*_ is the q-axis inductance is the flux linkage due permanent magnets. *w*_*e*_ is the electrical speed and *w*_*r*_ is the motor mechanical speed. After substituting by Eqs. (36) in (37) we get Eq. (38) shown below which expresses the current variation in both direct and quadrature rotating axes.

38 $$\left\{\begin{array}{l}\frac{d{i}_{d}}{dt}=-\frac{{r}_{s}}{{L}_{d}}{i}_{d}+p{w}_{r}{i}_{q}+\frac{{V}_{d}}{{L}_{d}}\\ \frac{d{i}_{q}}{dt}=-\frac{{r}_{s}}{{L}_{q}}{i}_{q}-p{w}_{r}{i}_{d}-\frac{p{w}_{r}\varphi }{{L}_{q}}+\frac{{V}_{q}}{{L}_{q}}\end{array}\right.$$

The electromagnetic torque developed by the PMSM is given by Eq. (39) shown below. It is worth noticing that rotor's rotation produces a mechanical torque given by Eq. (40) shown below where *T*_*e*_ is the electromagnetic torque and *T*_*r*_ is the mechanical load torque. *J* is the inertia of the rotor (Kg m^2^) and B is the mechanical damping coefficient. PMSM parameters used in this work are shown in Table 3.

**Table 3:** EV and environment parameters.

Parameter	Value
Weight	435 kg
Width	1.5 m
Heigth	1.459 m
Wheel radius	0.2 m
Maximum power	15 kW
Maximum torque	110 N m
Maximum speed	90 km/h
Acceleration constant	5 s
Air density	1.2 kg/m^3^
Aerodynamic coefficient	0.3

39 $${T}_{e}=(\frac{3p}{2})({\lambda }_{d}{i}_{q}-{\lambda }_{q}{i}_{d})$$

40 $${T}_{e}-{T}_{r}=J\frac{d{w}_{r}}{dt}+B{w}_{r}$$

**Figure 8:**
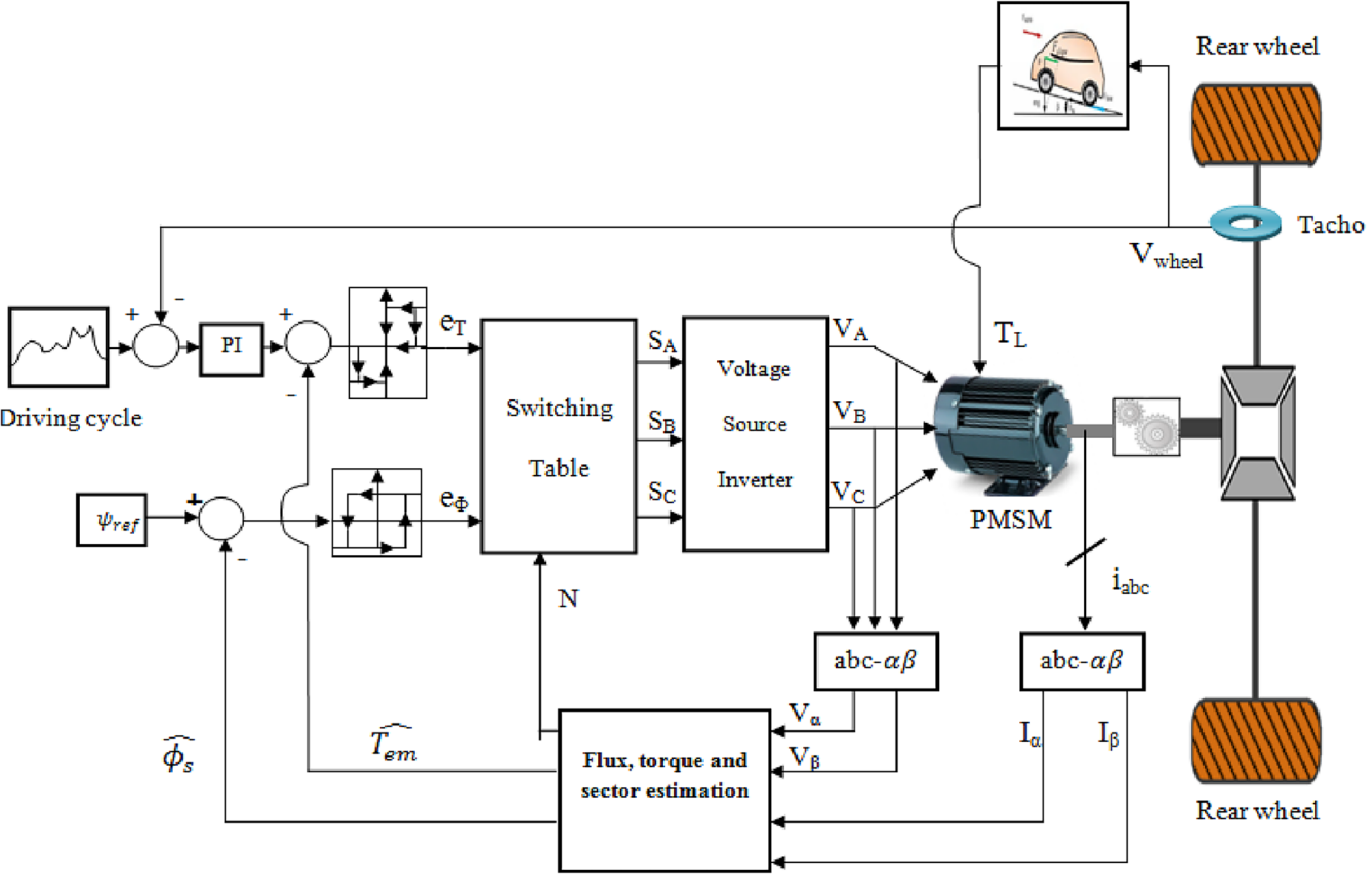
DTC electric vehicle control system.

The vector control technique known as DTC was initially put out in the 1980s by Takahashi and M. Depenbrock. This control strategy's basic method is to choose appropriate voltage vectors from the switching table according to the torque and flux hysteresis controllers' outputs, that will maintain the input error within a permitted error band. The direct torque control method used in an electric vehicle is depicted in the illustration below in Fig. 8.

The stator flux is estimated using the Eq. (41) shown below where *r*_*s*_ is the stator resistance and *V*_*s*_ is the stator voltage which is expressed in *αβ* frame using Eq. (42) shown below:

41 $${\phi }_{s}(t)={\phi }_{PM}+{\int }_{0}^{t}({V}_{s}(t)-{r}_{s}{i}_{s}(t))dt$$

42 $${V}_{s}={V}_{s\alpha }+j{V}_{s\beta }$$

Integration of the difference between the input stator voltage and the voltage drop across the stator resistance in *αβ* frame yields the set of equation given in (43) where $\varphi_{PM}$ the permanent magnet flux.

43 $$\left\{\begin{array}{c}{\phi }_{s\alpha }(t)={\phi }_{PM}+{\int }_{0}^{t}({V}_{s\alpha }(t)-{r}_{s}{i}_{s\alpha }(t))dt\\ {\phi }_{s\beta }(t)={\int }_{0}^{t}({V}_{s\beta }(t)-{r}_{s}{i}_{s\beta }(t))dt\end{array}\right.$$

The estimated stator flux and angle are derived from Eq. (43) and they are respectively given by Eqs. (44) and (45) shown below:

44 $$\stackrel{\wedge }{{\varphi }_{s}}=\sqrt{{\varphi }_{s\alpha }^{2}+{\varphi }_{s\beta }^{2}}$$

45 $$\stackrel{\wedge }{{\theta }_{s}}={\mathit{tan}}^{-1}\left(\frac{{\varphi }_{s\beta }}{{\varphi }_{s\alpha }}\right)$$

**Figure 9:**
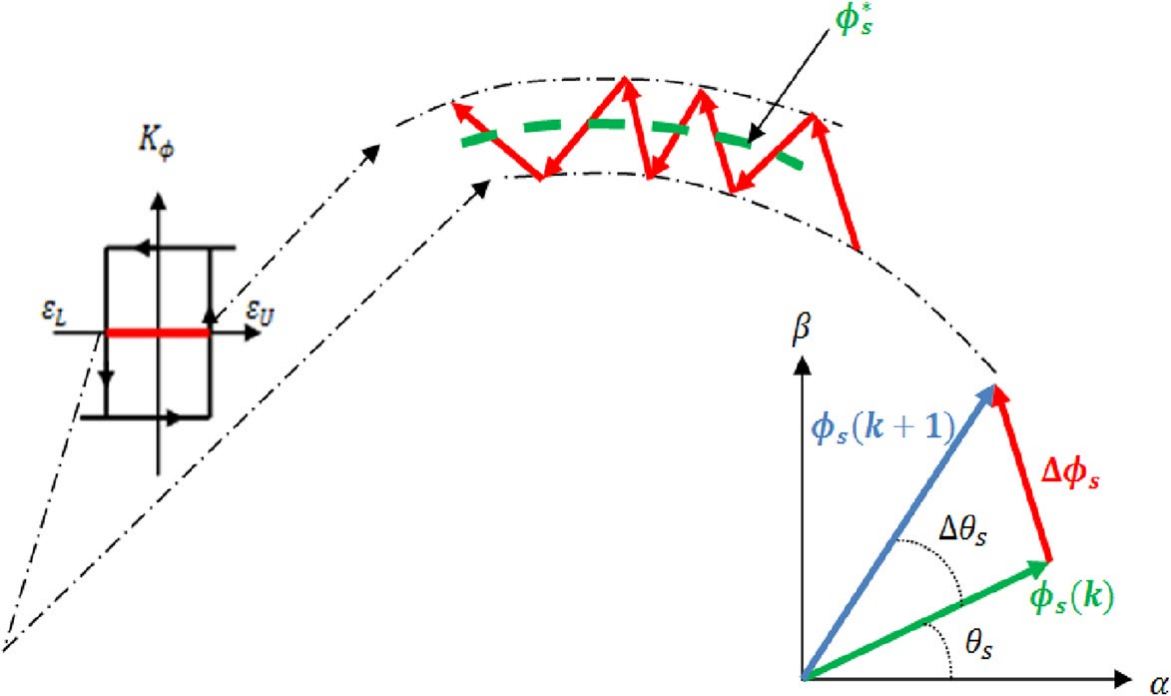
Stator flux vector evolution in the αβ subspace.

For managing flux, we utilize both two-level and three-level hysteresis controllers. The hysteresis flux controller evaluates the stator reference flux against its estimated counterpart. If the flux error surpasses the upper hysteresis limit ε_U_, the controller issues an output of 1; conversely, if the flux error falls below the lower hysteresis limit ε_L_, the output is set to 0. In torque control, the hysteresis controller follows a similar pattern. If the estimated torque exceeds the reference value, the controller outputs − 1; for torque values below the reference torque, the output is 1. Meanwhile, the output is set to 0 for estimated torque values falling between the lower and upper torque hysteresis bands. Figure 9 shows the stator flux vector evolution in the αβ subspace.

## Power management and control

**Figure 10:**
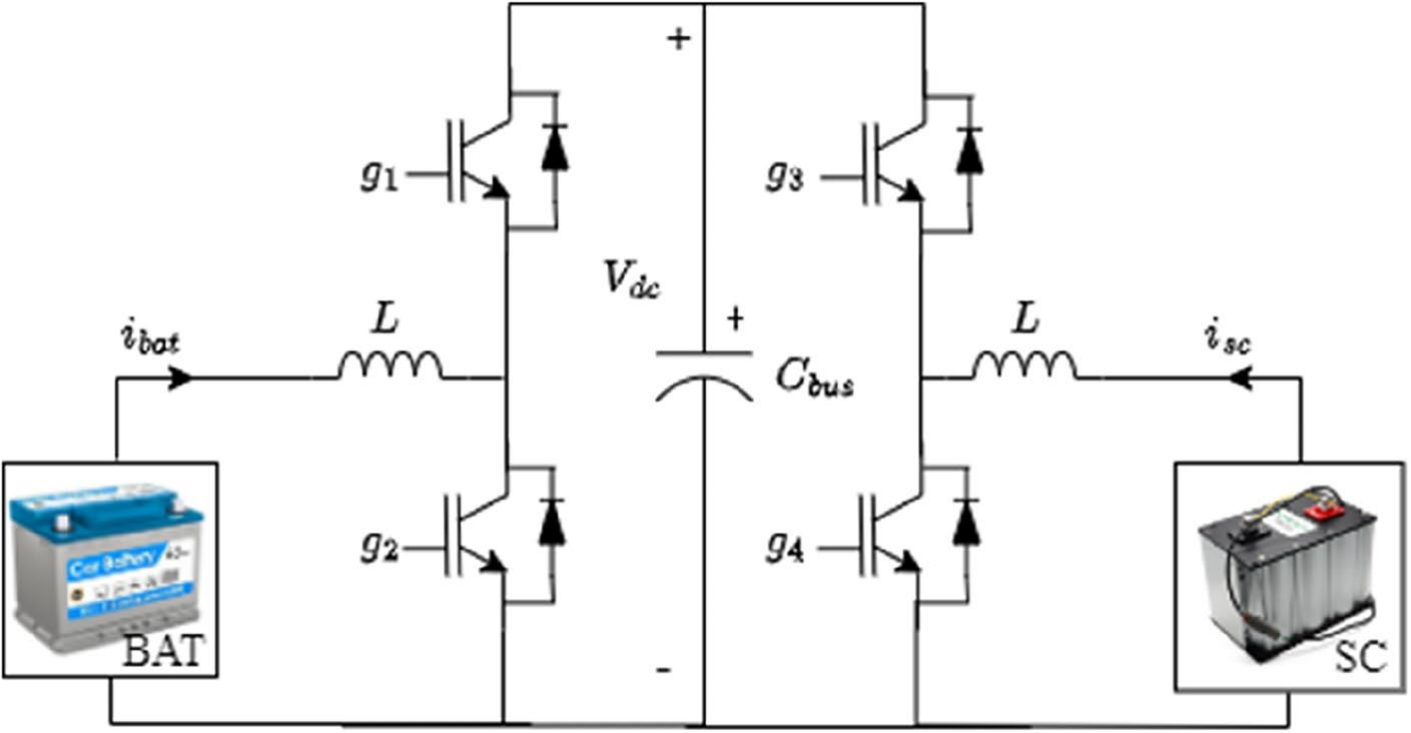
Connection of power sources to DC bus.

**Figure 11:**
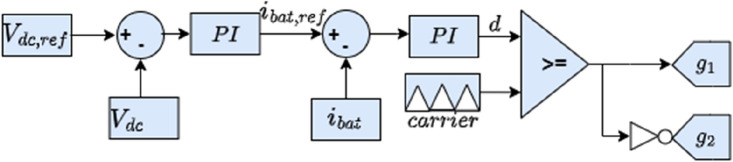
Battery control loop.

**Figure 12:**
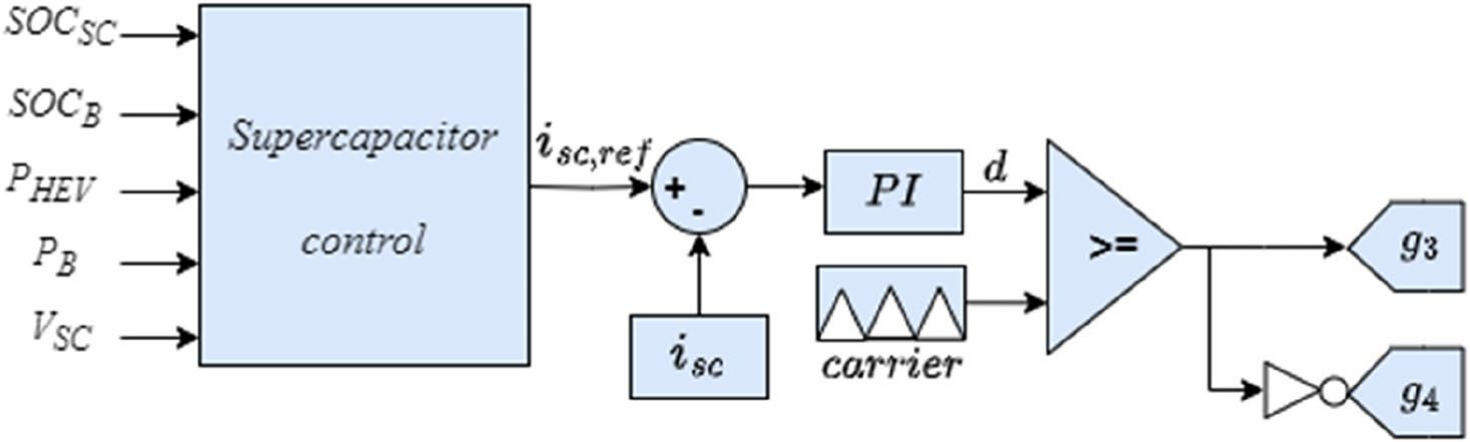
Supercapacitor control loop.

Two bidirectional DC–DC converters are used for the connection of the battery and supercapacitor to the DC bus as shown in Fig. 10. This will allow energy recovery during regenerative breaking periods which will increase the autonomy of the HEV. The battery fixes the DC bus voltage at its reference value using the control loop shown in the Fig. 11 where two PI controllers are used in cascade to produce a variable duty cycle d. This last-mentioned quantity will be continuously compared to a carrier signal to produce the gate signals g1 and g2 which will trigger the converter IGBTs. In this work, the SC is used mainly during HEV accelerations, high power demands and in situations during which the battery SOC is relatively low with respect to SOC_SC_. Otherwise, the SC will be turned off. The control loop used to regulate the SC current is shown in Fig. 12. One can notice from the same figure that the SC current is a function of five distinct variables.

In this work, the SC is used mainly during HEV accelerations, high power demands and in situations during which the battery SOC is relatively low with respect to SOC_SC_. Otherwise, the SC will be turned off. The control loop used to regulate the SC current is shown in Fig. 6. One can notice from the same figure that the SC current is a function of five distinct variables.

The required power for traction, P_HEV_, is divided into three sets: High power P_H_, Average power P_A_ and low power P_L_. The state of charge of both power sources is divided into four levels: Full (F), Average (A), Almost Low (AL) and Low (L) as it is shown in Eq. (46). The numerical values chosen for state of charge levels were based on a combination of expected operating ranges for the battery and supercapacitor, as well as industry standards and guidelines extracted from literature and published papers (see the references just below this paragraph). Based on the aforementioned references and recommendations, we have choosed to choose the interval [80%, 90%] for the fully state of charge level, [65–80%] for average state of charge level, [30%, 65%] for almost average state of charge level. Finally, the range from 00 to 65% is used for low state of charge level.

46 $$SO{C}_{SC,BAT}=\left\{\begin{array}{l}F;80\le SO{C}_{SC,BAT}\le 90\\ A;65\le SO{C}_{SC,BAT}\prec 80\\ AL;30\le SO{C}_{SC,BAT}\prec 65\\ L;0\le SO{C}_{SC,BAT}\prec 30\end{array}\right.$$

**Figure 13:**
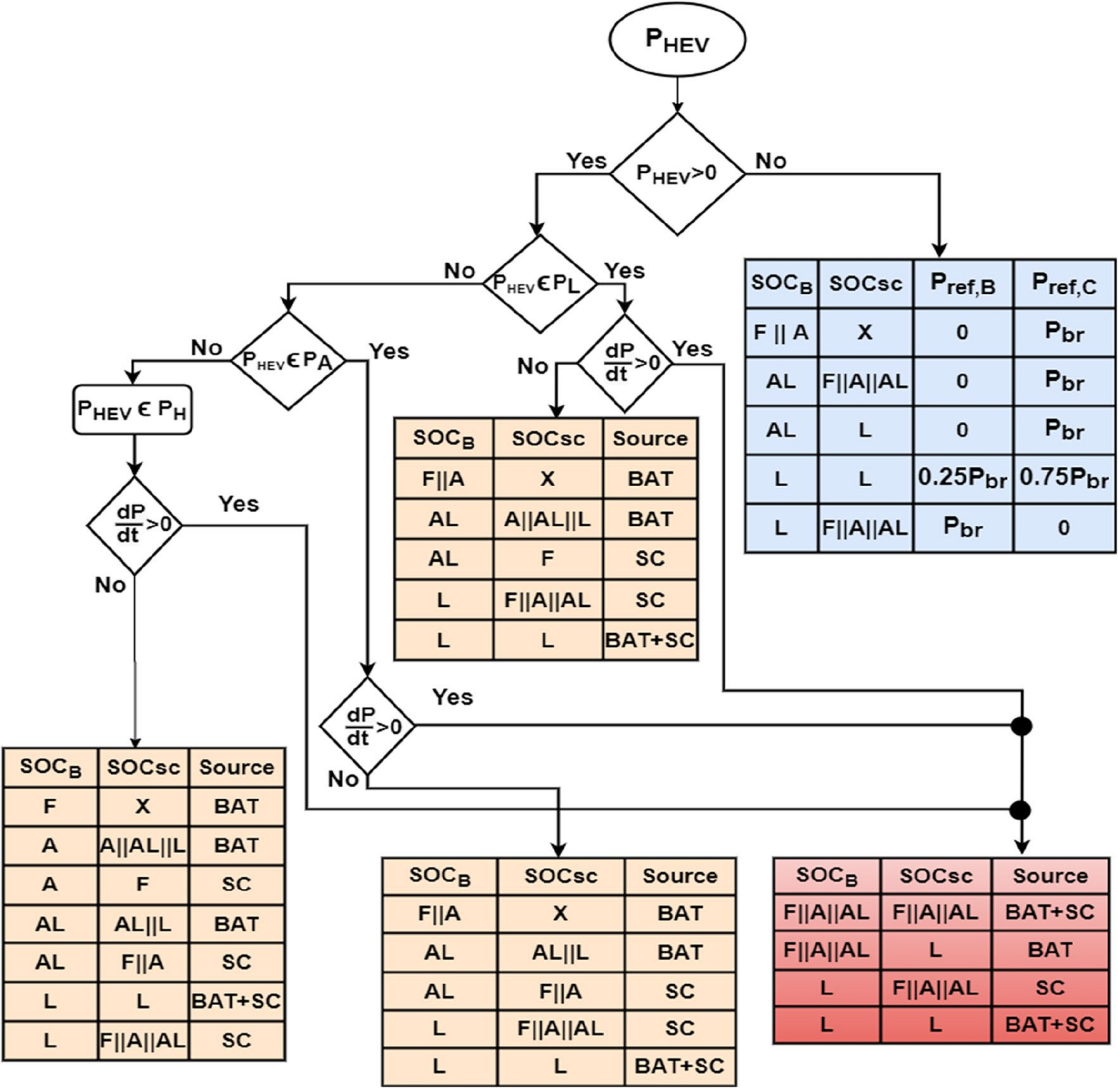
Power management algorithm.

A power management algorithm was developed to provide an effective usage of vehicle power sources which will increase their lifetime and improve vehicle autonomy. The flowchart shown in Fig. 13 explains the major steps of the proposed power management algorithm. The ‘*X*’ mark on Fig. 13 represent a don’t care condition. The following remarks could be made about the flowchart in Fig. 13:

- During increasing power demands, the decision about the source to be used doesn’t depend on the level of the required power. It does depend on the SOC of power sources.
- During increasing power demands, the supercapacitor operates either alone or in parallel with the battery depending on the SOC of these two sources. If the supercapacitor SOC is low then the battery will be used even in cases of increasing power demands.
- During constant traction mode, the decision about the source to be used depends on the SOC of power sources and on the level of the required power for traction.
- During constant traction mode, the battery is used to ensure HEV traction except in cases where its state of charge is low relatively to supercapacitor SOC.

Most of times, the energy recovered during regenerative braking will be used to charge the supercapacitor. This is because the battery does not sustain high and abrupt currents. Also, charging the battery during braking periods is not really a clever idea because of its slow dynamics and its high charging time compared to supercapacitors. Some exceptions are made. For instance, when the supercapacitor and battery SOC are both low, 75% of the recovered energy will be used to charge the supercapacitor and 25% will be used for charging the battery.

**Figure 14:**
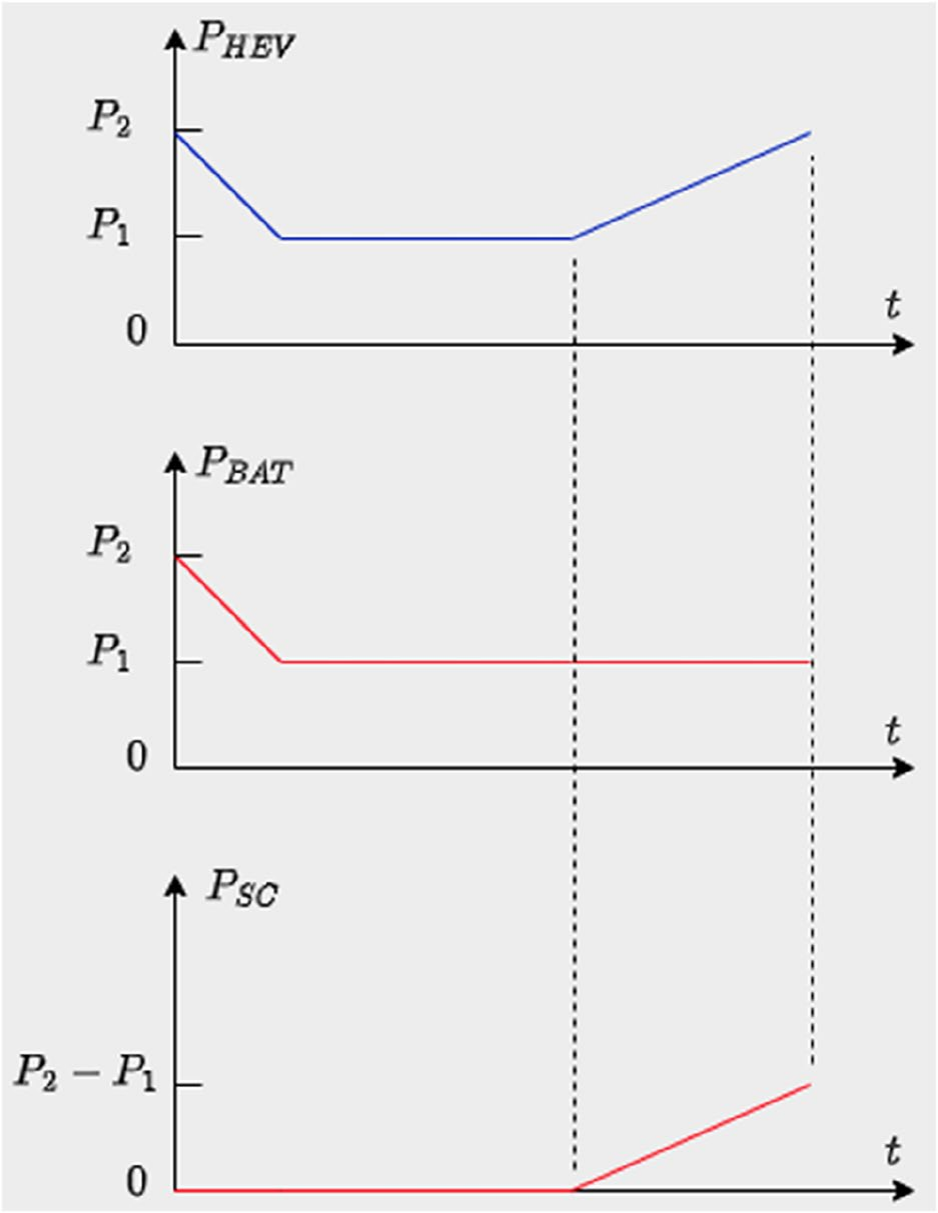
Simultaneous SC and BAT operation.

BAT and SC operations are illustrated in Fig. 14 shown below. The instant during which the power slope becomes strictly positive, the battery will continue to operate at a constant power equal to its value during the triggering instant and the supercapacitor will provide the missing power. Constant power operation will provide more safety, efficient operation and longer lifespan for the battery.

## Simulation and results

The effectiveness of the proposed coordinated switching strategy at both motor and power sources level is tested using numerical simulations under MATLAB/Simulink environment. Fixed step simulation using Runge–Kutta numerical solver is adopted in this work. The parameters used to conduct the different simulations that will be presented below and PMSM parameters are illustrated in the Tables 4 and 5, respectively.

**Table 4:** Different simulation parameters.

Symbol	Value
*T*_*S*_	1e−6 s
*T*_*TH*_	80 N m
*T*_*SWS*_	1 s
*n*	10
*m*	40
*T*_*SWM*_	25 ms

**Table 5:** PMSM parameters.

Parameter	Value
*P*_*n*_	35 kW
*T*_*n*_	111 N m
*r*_*s*_	0.05 Ohm
*L*	6.35 e−4 mH
*Φ*	0.192 Wb
*J*	0.011 kg m^2^
*B*	0.002 N m s
*P*	4

**Figure 15:**
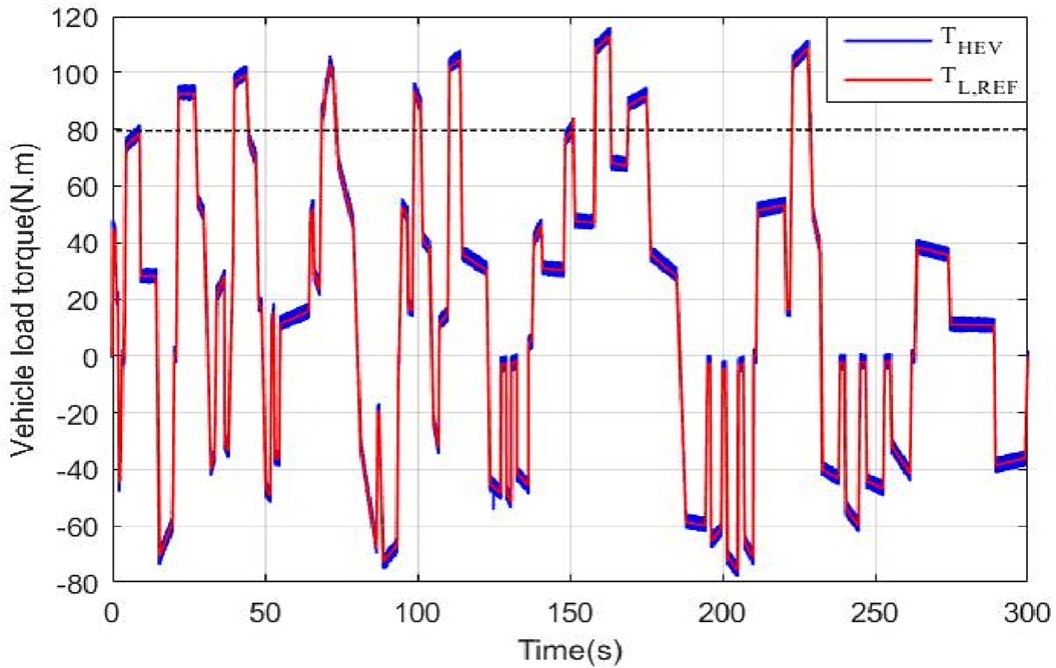
HEV torque and its reference.

**Figure 16:**
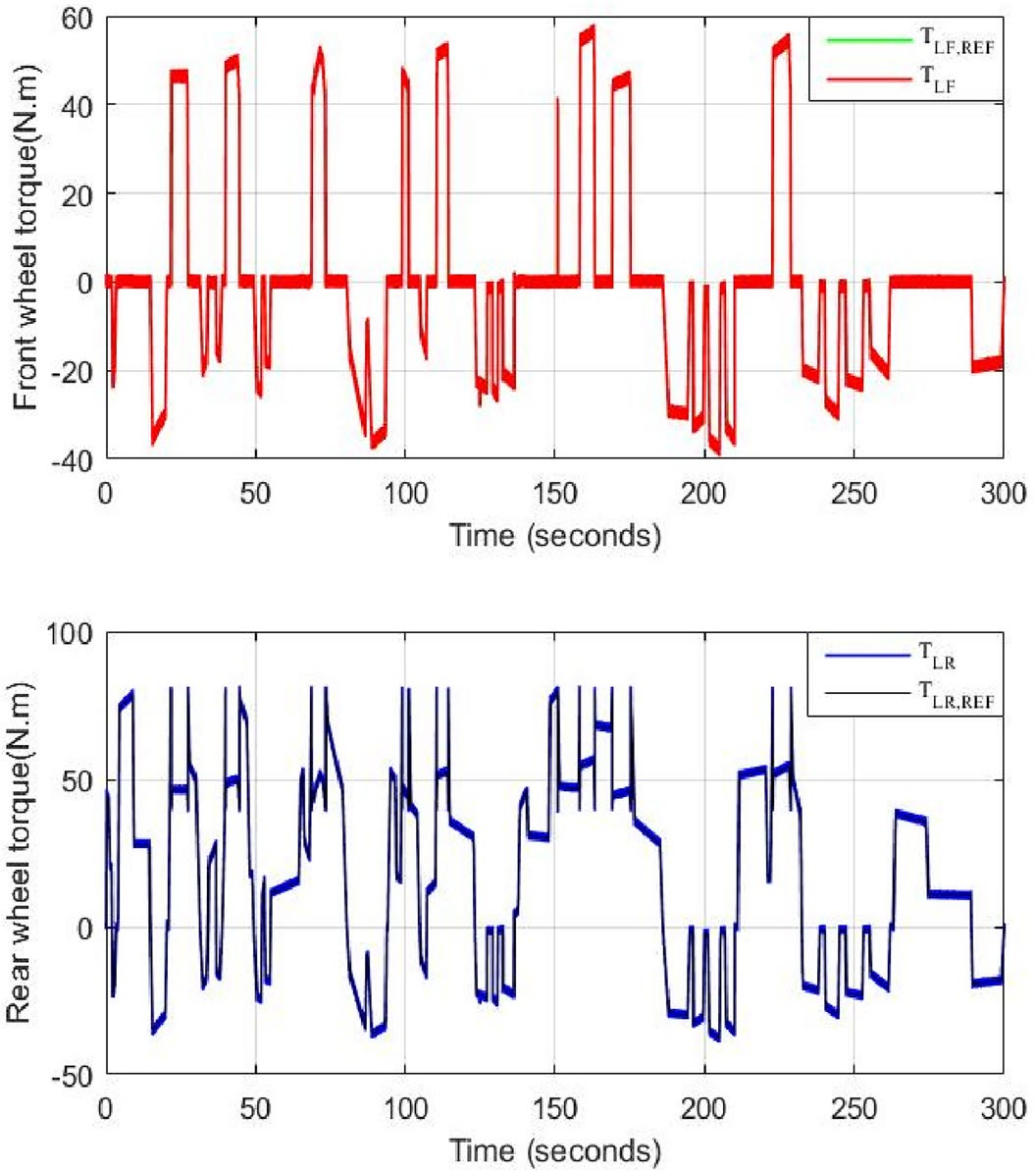
Front and rear wheel torque.

Figure 15 shows the torque of the HEV and its reference. It could be seen that the vehicle torque follows its reference. The discontinuous black lines highlight the threshold torque value above which the 4WD mode will be activated. Figure 16 shows the torque of the front and rear traction machines.

**Figure 17:**
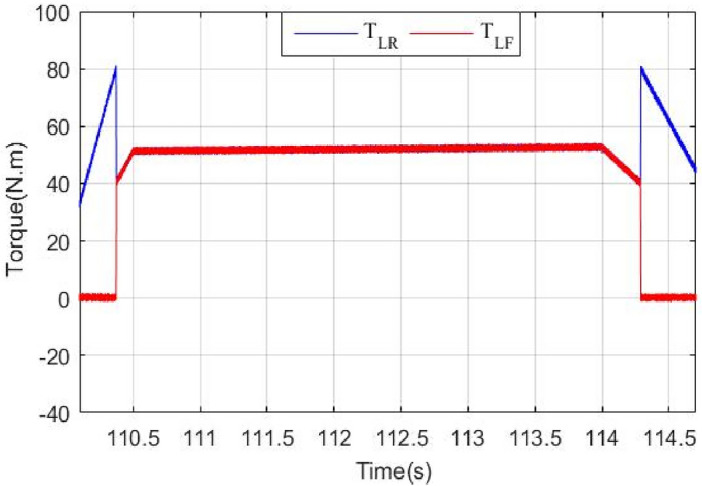
Zoom of front and rear wheel torque.

It could be noticed that when the torque is below 80 N m, the rear machine will ensure alone HEV traction. As soon as the load torque exceeds this threshold value, the four wheel drive mode (4WD) will be activated by connecting the front traction machine to the front wheels. In this case, both front and rear PMSM will ensure vehicle traction. This is well illustrated in Fig. 17.

**Figure 18:**
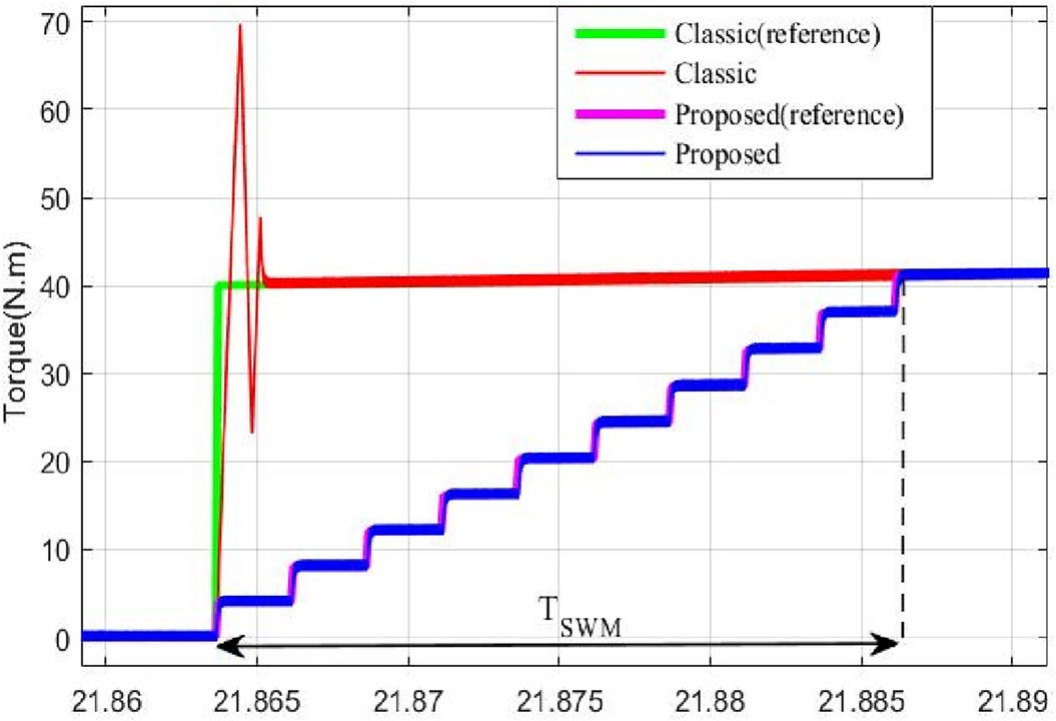
Front PMSM behavior during RWD to 4WD transition.

**Figure 19:**
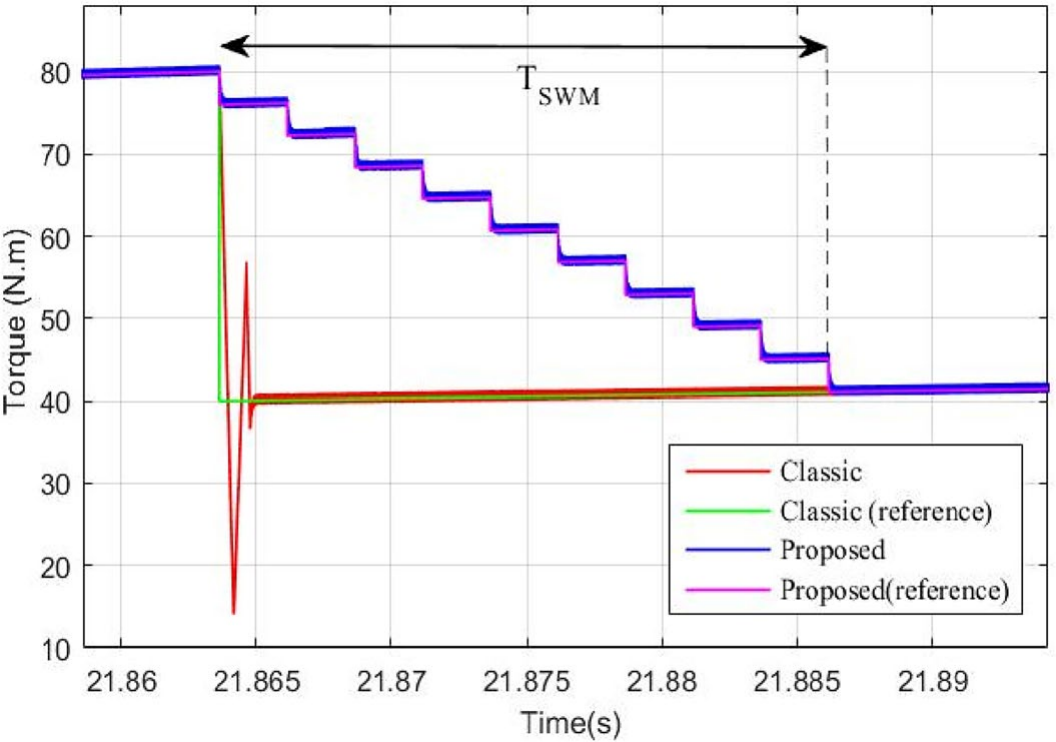
Rear PMSM behavior during RWD to 4WD transition.

Zooms showing the transition from RWD to 4WD and vice versa are shown in Figs. 18 and 19. One can note that thanks to the proposed commutation algorithm, no torque ripples are observed during transitions between the different traction modes. In order to point out the improvements brought by the proposed commutation technique, a comparison to classical or binary commutation strategy is performed. Binary commutation strategy stands for instantaneous turn off and turns on of traction machines without passing through a transient period. One can conclude that the proposed commutation algorithm has succeeded to suppress the undesired ripples which are very harmful for traction machines and can cause serious damages.

**Figure 20:**
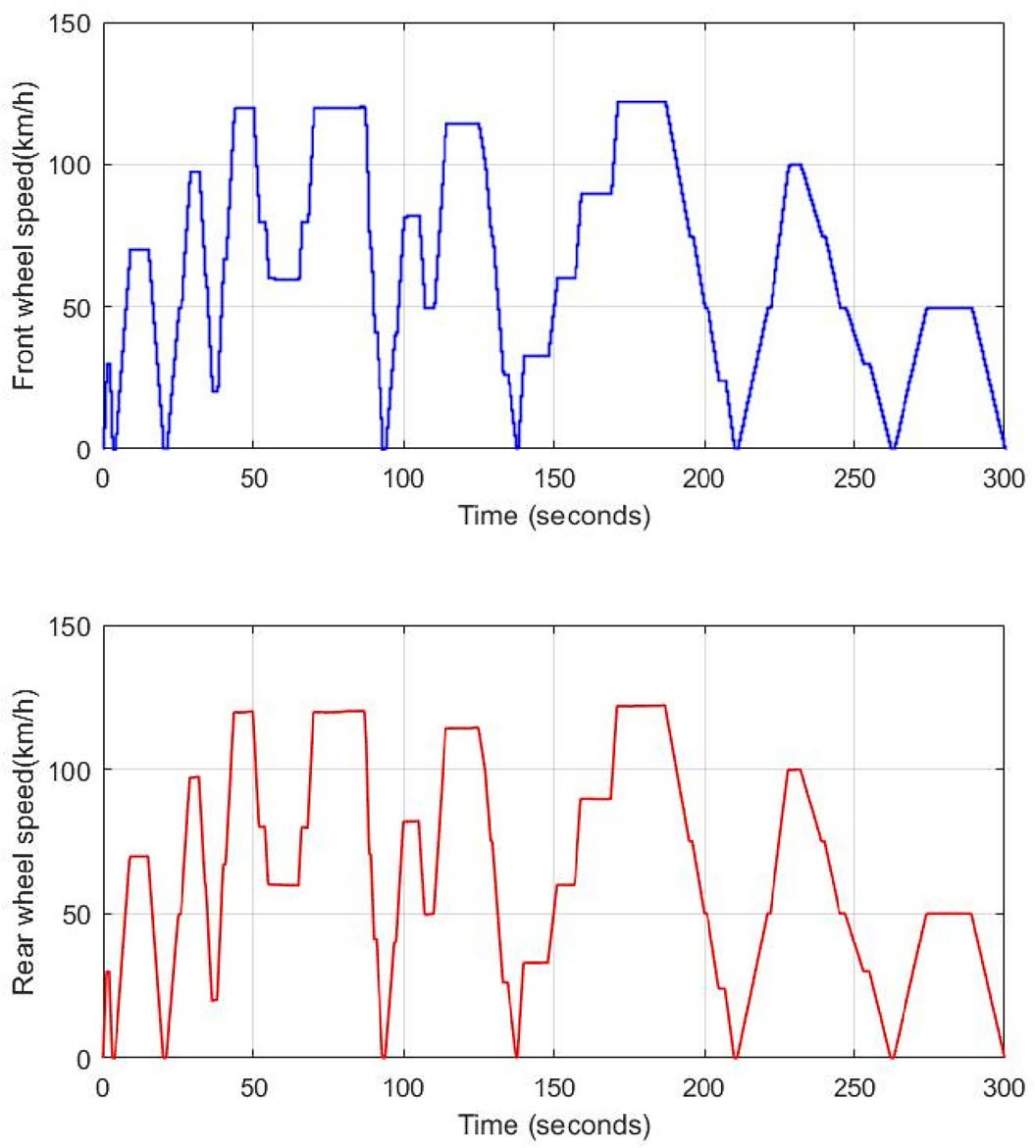
Front and rear HEV wheel speed.

**Figure 21:**
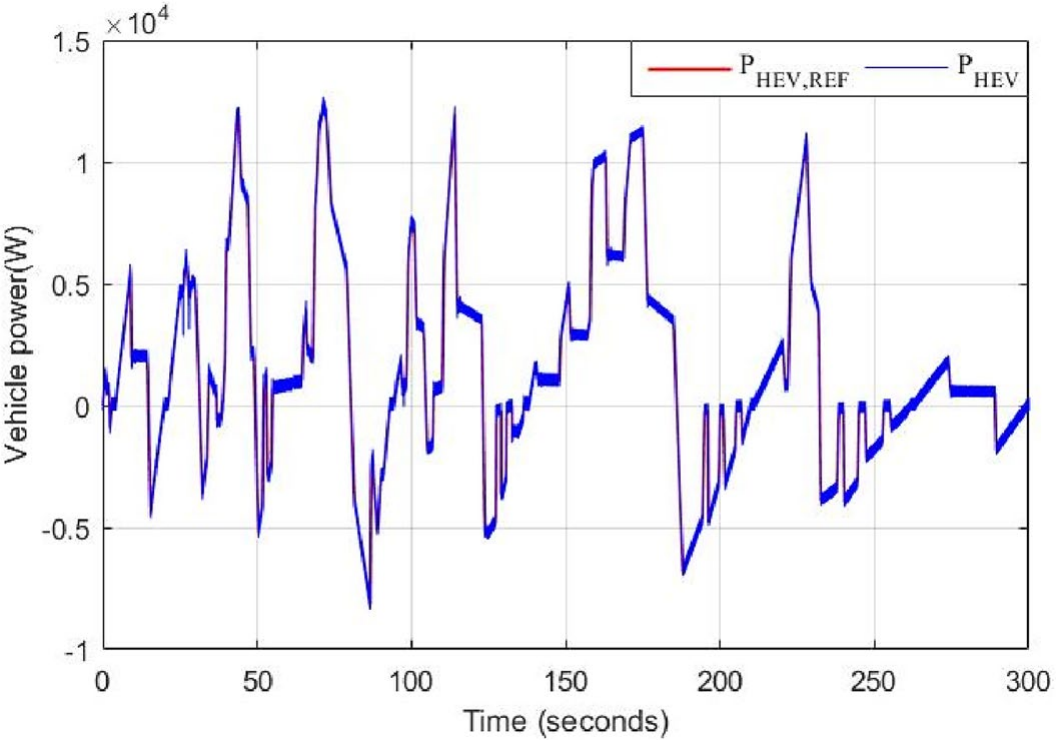
HEV traction power and its reference.

The speed of front and rear wheels is shown in Fig. 20. It is worth noticing that the front wheel is either driving (4WD) or driven (RWD) depending on the traction mode. In both cases, all HEV wheels have the same speed and no significant speed ripples are noticed during RWD/4WD or vice versa commutations. The HEV power is shown in Fig. 21. One can see that HEV tracks well its reference.

**Figure 22:**
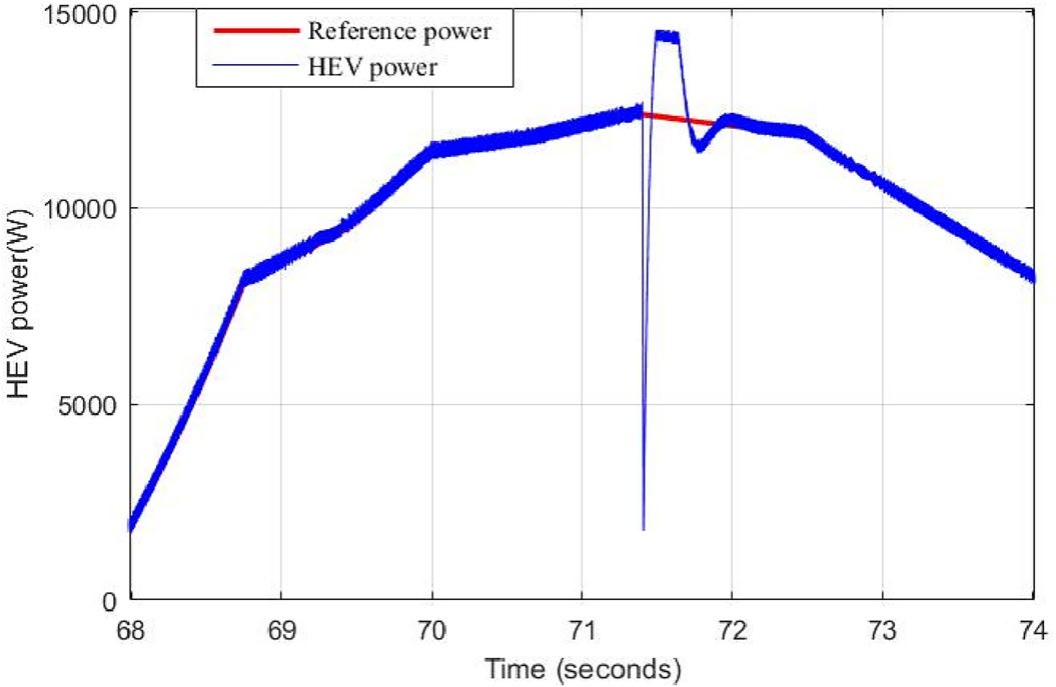
Power ripples due to classical switching.

**Figure 23:**
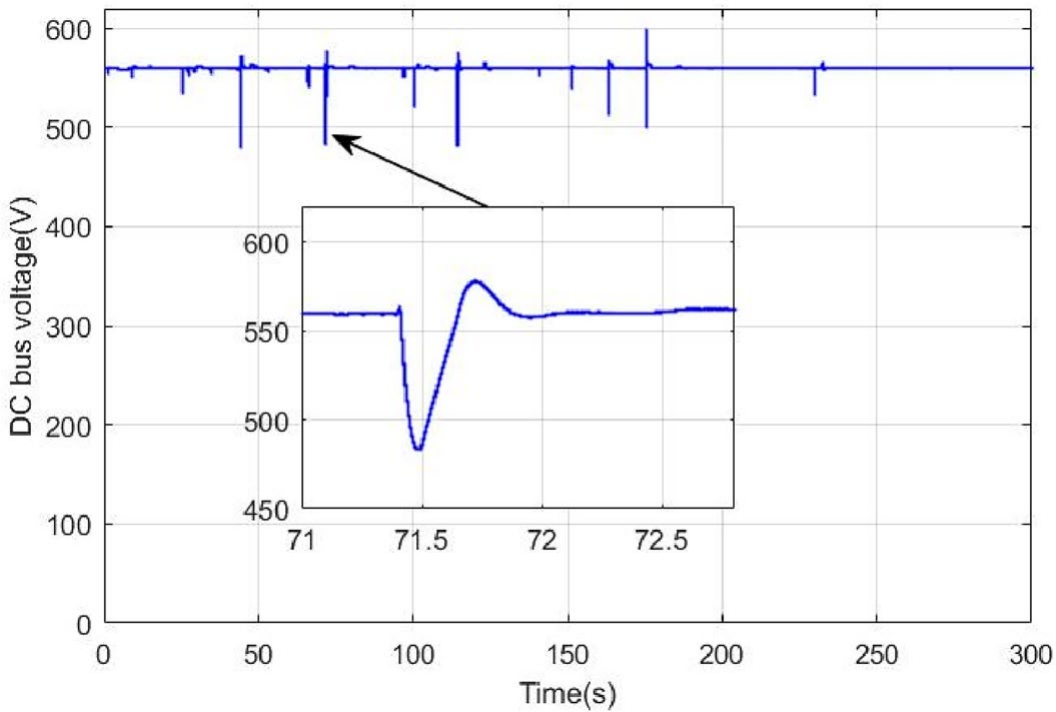
DC bus voltage using classical switching.

Battery and supercapacitor powers are depicted in Figs. 22 and 23 respectively. Note that the battery is rarely charged during regenerative braking periods and this is because of its slow dynamics and its limited number of charge/discharge cycles compared to supercapacitors. In this work, this last-mentioned power source is mostly used to store energy during regenerative braking periods.

In order to improve HEV autonomy and to ensure driving comfort, switching between power sources is necessary. Generally switching is performed used binary logic approach which is defined by the following piece wise function

47 $${P}_{source}=\left\{\begin{array}{ccc}{P}_{ref}& when& s=1\\ 0& when& s=0\end{array}\right.$$

The equation above means that a given power source is *off* when its corresponding control signal s is null and it is instantaneously *tuned on* as soon as its control signal goes high. This type of switching results in significant peaks of power and large DC bus voltage ripples. To illustrate this fact, the classical switching method is compared to the proposed switching strategy.

When the supercapacitor is switched off at 71.4 s, the binary switching method has instantaneously set the supercapacitor reference power to zero. Hence, the battery, known by its slow dynamics, has to deliver an important amount of power in a very small-time interval. This will produce important power peaks as it is depicted in Fig. 22. Large and abrupt peaks can cause serious damages in power sources. Another drawback of this classical way of switching is pointed out in Fig. 23 where one can see that the binary switching resulted in large DC bus ripples (up to 100 V). This is very risky in HEV applications because if DC bus regulation is lost then no power can be supplied to traction motors.

**Figure 24:**
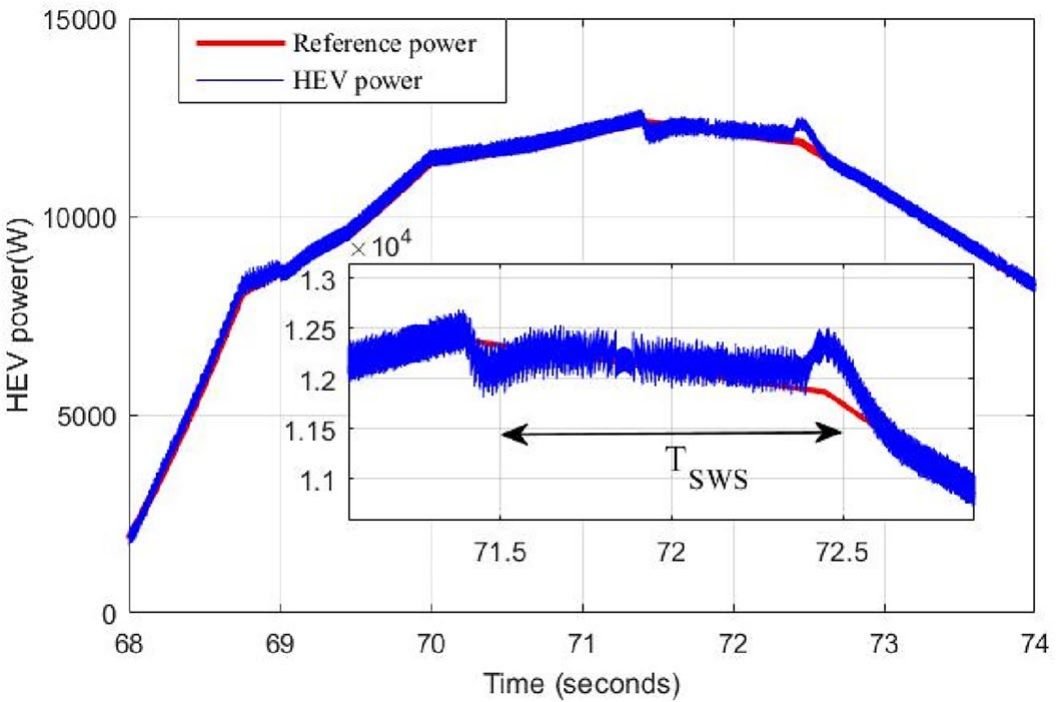
Power ripples due to proposed switching strategy.

To overcome the problem of ripples when switching between power sources. The proposed gradual switching strategy is applied to the HEV system. Figure 24 shows the HEV power when the supercapacitor is turned off at t = 71.4 s. Note that the gradual extinction made in T_SWS_ seconds resulted in the suppression of almost all power ripples during the transition.

**Figure 25:**
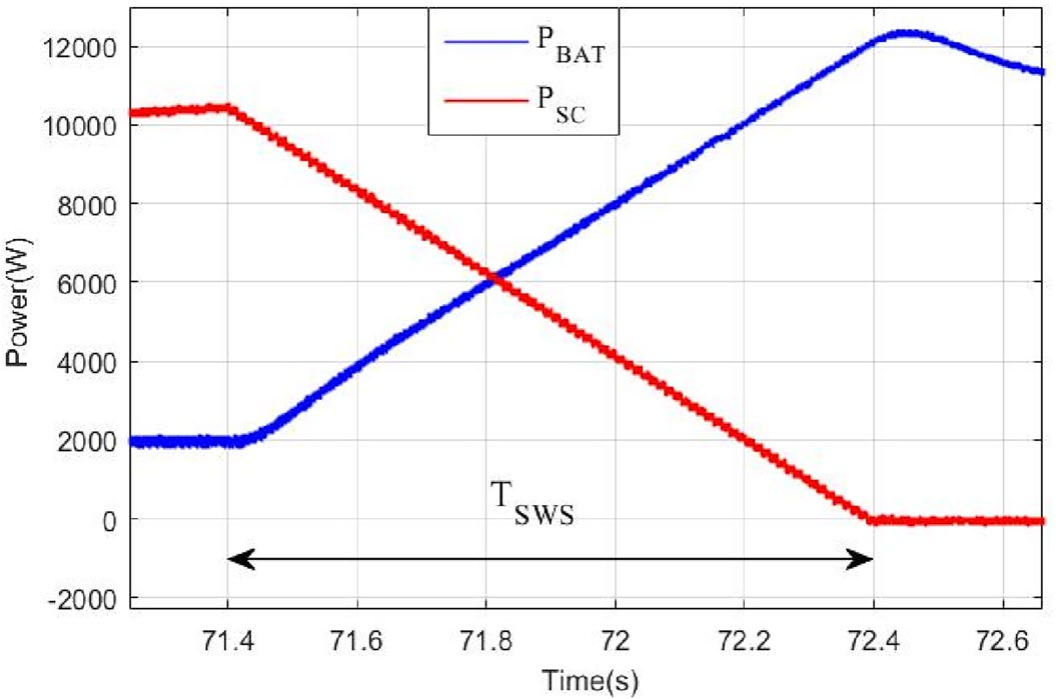
DC bus voltage using proposed coordinated switching strategy.

**Figure 26:**
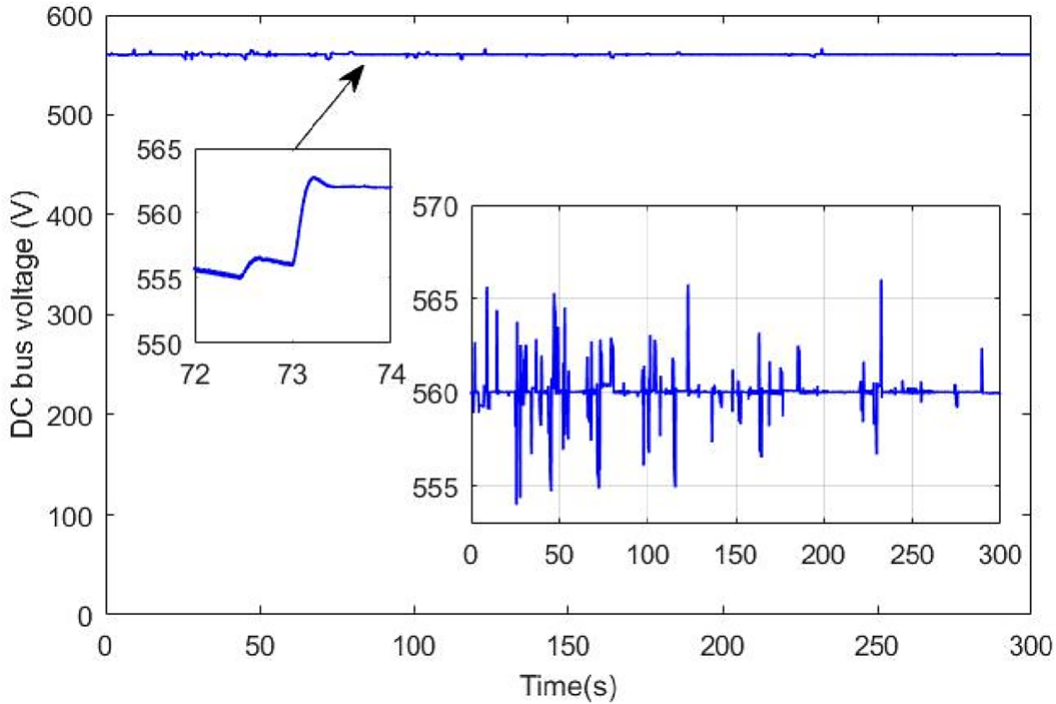
DC bus ripples due to classical switching.

Figure 25 shows the how the supercapacitor is gradually turned off using the proposed switching strategy. This progressive turns off gave enough time to the battery known by its slow dynamics to reach the reference power within T_SWS_ second. Figure 26 shows the DC bus voltage when gradual switching technique is used. One can conclude that the application of this technique has resulted in significant minimization of voltage ripples and has enhanced DC link voltage stability. Note that the maximum absolute voltage deviation is around 6 V which represents 1% of the nominal value.

**Figure 27:**
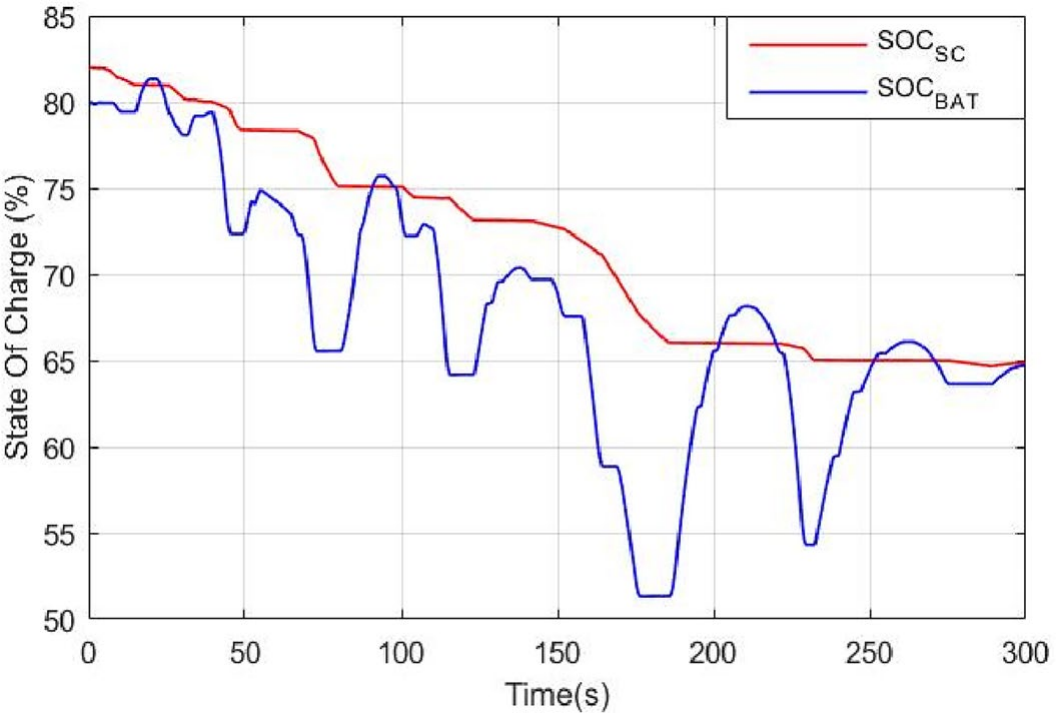
Battery and supercapacitor state of charge.

Figure 27 shows the battery and supercapacitor state of charge. The first remark that could be made is that both power sources started almost with the same SOC and they ended nearly with the same SOC value. This reflects the efficiency of the used power management that ensured moderate use of power sources and avoided exhausting one source over another. The supercapacitor is used during regenerative braking because of its fast dynamics and its ability to sustain high and abrupt currents occurring.

## Real-time validation

**Figure 28:**
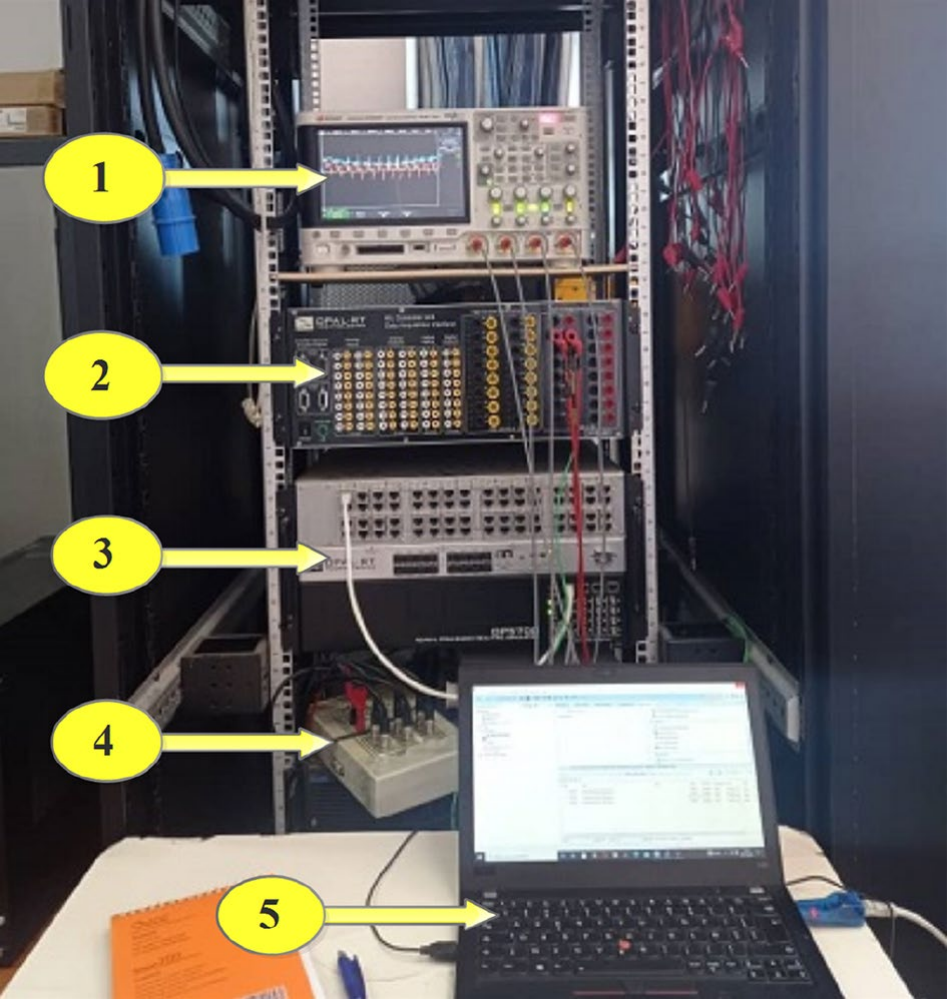
RT LAB test bench.

**Figure 29:**
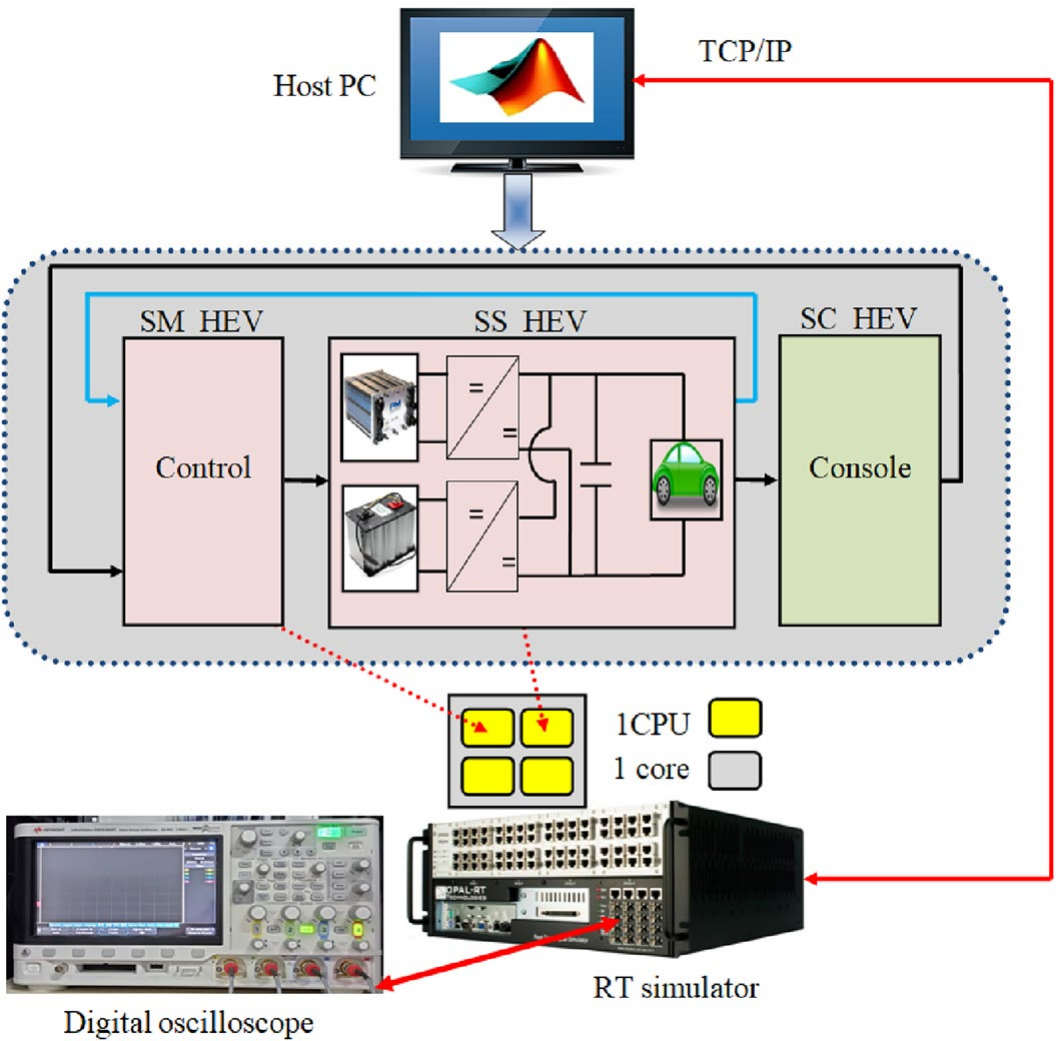
RT LAB real time simulation setup.

The proposed coordinated switching strategy undergoes real-time validation using the OPAL RT simulation platform. In Fig. 28, our research laboratory's real-time simulation bench setup is depicted. Element 1 signifies a digital oscilloscope, while element 2 denotes a unit measurement data acquisition interface (OP8660). Element 3 illustrates the FPGA-based real-time simulator (OP 5700), and element 4 represents an isolation and amplification card. Finally, element 5 corresponds to the host PC. Figure 29 depicts how the developed MATLAB model is separated into master, slave and console blocks as a first step prior its real time simulation on RT LAB platform. SM_HEV in the last-mentioned figure represents the master computational block that contains the developed coordinated switching strategy discussed in this paper. The control loops of the DC-DC converter associated with the used power sources are also included in the master computational block. Battery, supercapacitor and converter models are incorporated in the slave calculating block (SS_HEV). The execution of master and slave computational blocks occurs on separate CPU cores of the OPAL RT simulator, as depicted in Fig. 29. Data exchange between the computing subsystem and the GUI subsystem or a console takes place asynchronously via the TCP/IP link. However, synchronous handling of data interchange between the two computation subsystems is facilitated through shared memory.

**Figure 30:**
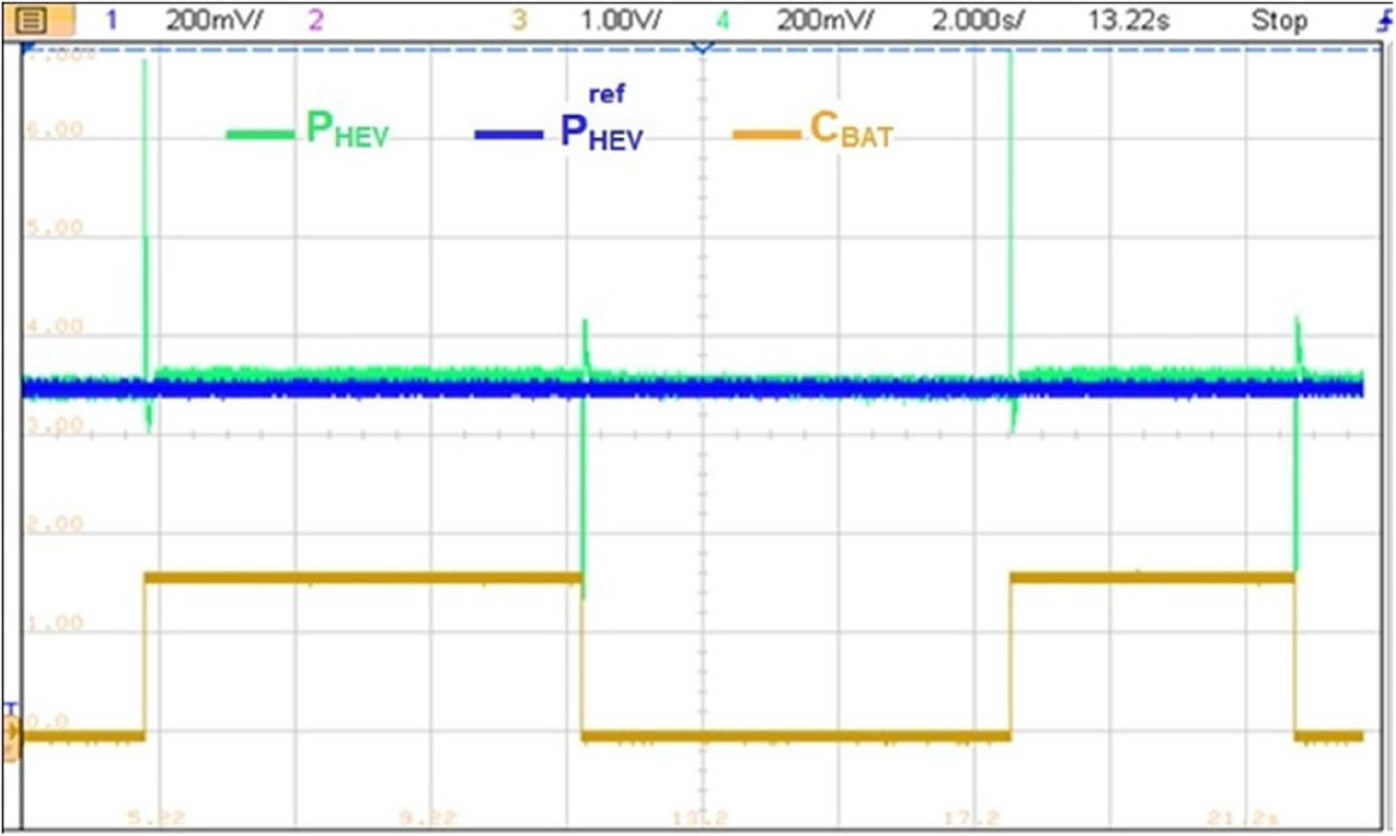
Transient peaks due to abrupt power source switchings.

The reference HEV power is set to 7 kW (P_HEV_ = 7 kW) as it is highlighted in yellow on Fig. 30. This last power value was obtained after the multiplication of the 3.5 divisions times 200 mV of each division times the 10 k which is the attenuation and protection scale of the RT LAB. The blue signal on the same figure represents the BAT power contribution factor C_BAT_ and the red represents the sum of SC and BAT developed power. One can remark that developed HEV power follows its reference but large ripples are observed during each C_BAT_ toggling. These ripples are due to miscoordination between BAT and SC transient dynamics.

**Figure 31:**
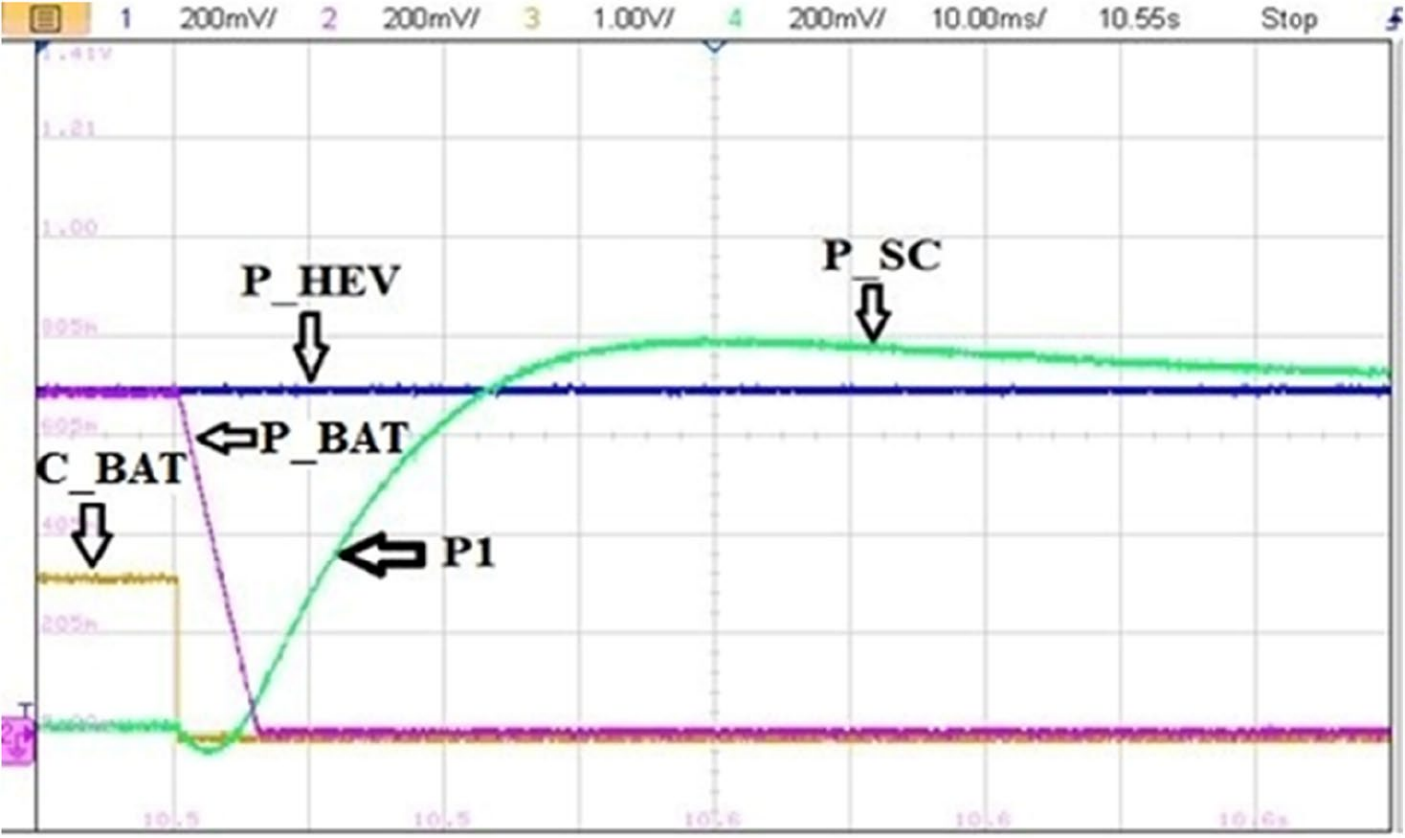
Non coordinated power source switchings.

Figure 31 illustrates a non-coordinated switching between BAT and SC. Abrupt BAT switching caused a transient power lack as it is the case at point P1 in Fig. 33 where the developed power by BAT and SC is only about half the required power for traction. This will affect significantly the vehicle riding comfort and performance.

**Figure 32:**
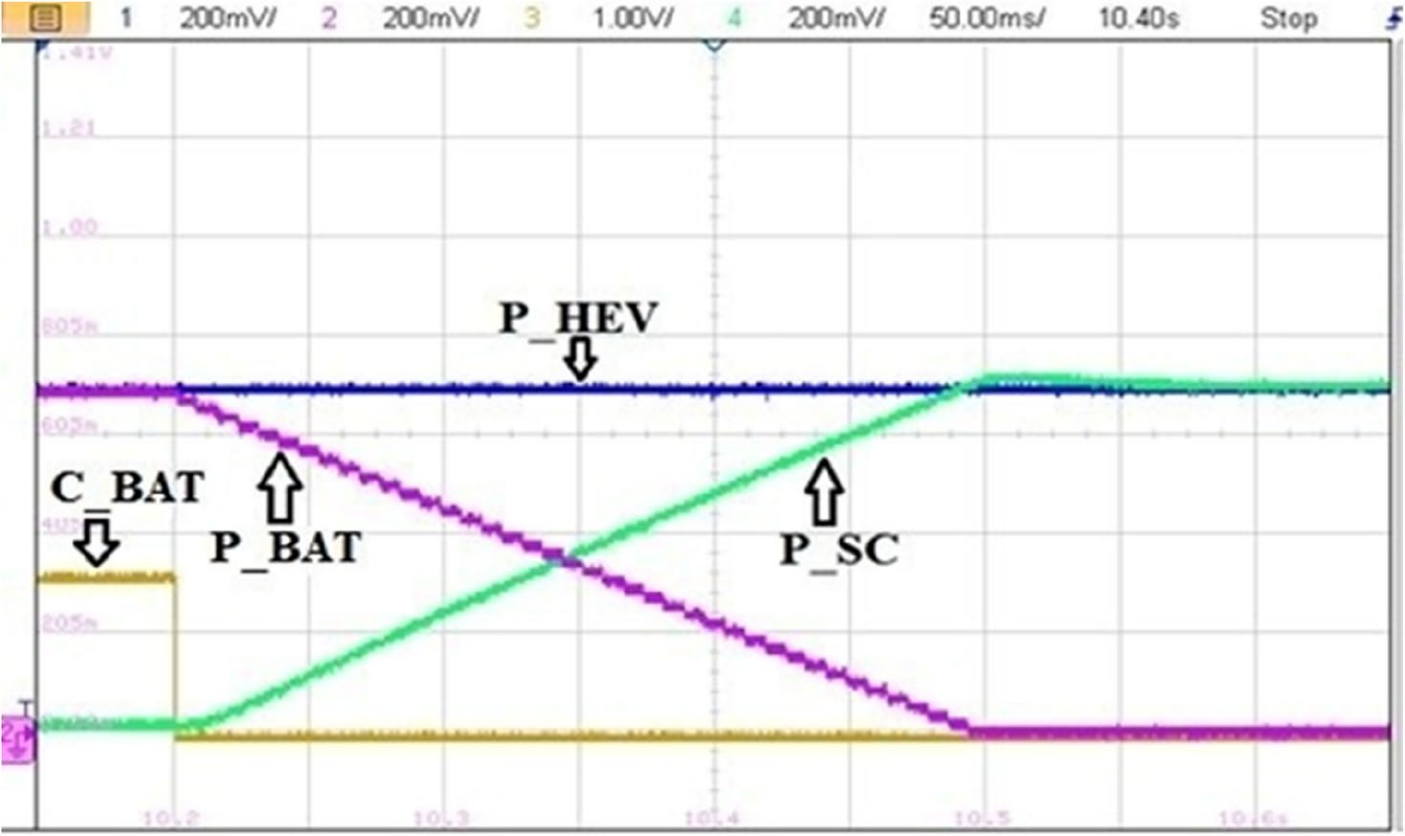
Coordinated power source switchings.

Figure 32 highlights a zoom of coordinated BAT-SC switching. From this figure, one can see that when C_BAT_ toggles from 1 to 0, BAT power is set to zero via the stair-based transition function. The used transition function has made the sum of BAT and a SC power at every point during the transition is equal to the reference HEV power. This will enhance driving comfort and will suppress the transient ripples noticed in abrupt switching. Furthermore, this transition strategy enhances also power sources lifetime since it prevents drawing abrupt high currents that may cause power source damage.

**Figure 33:**
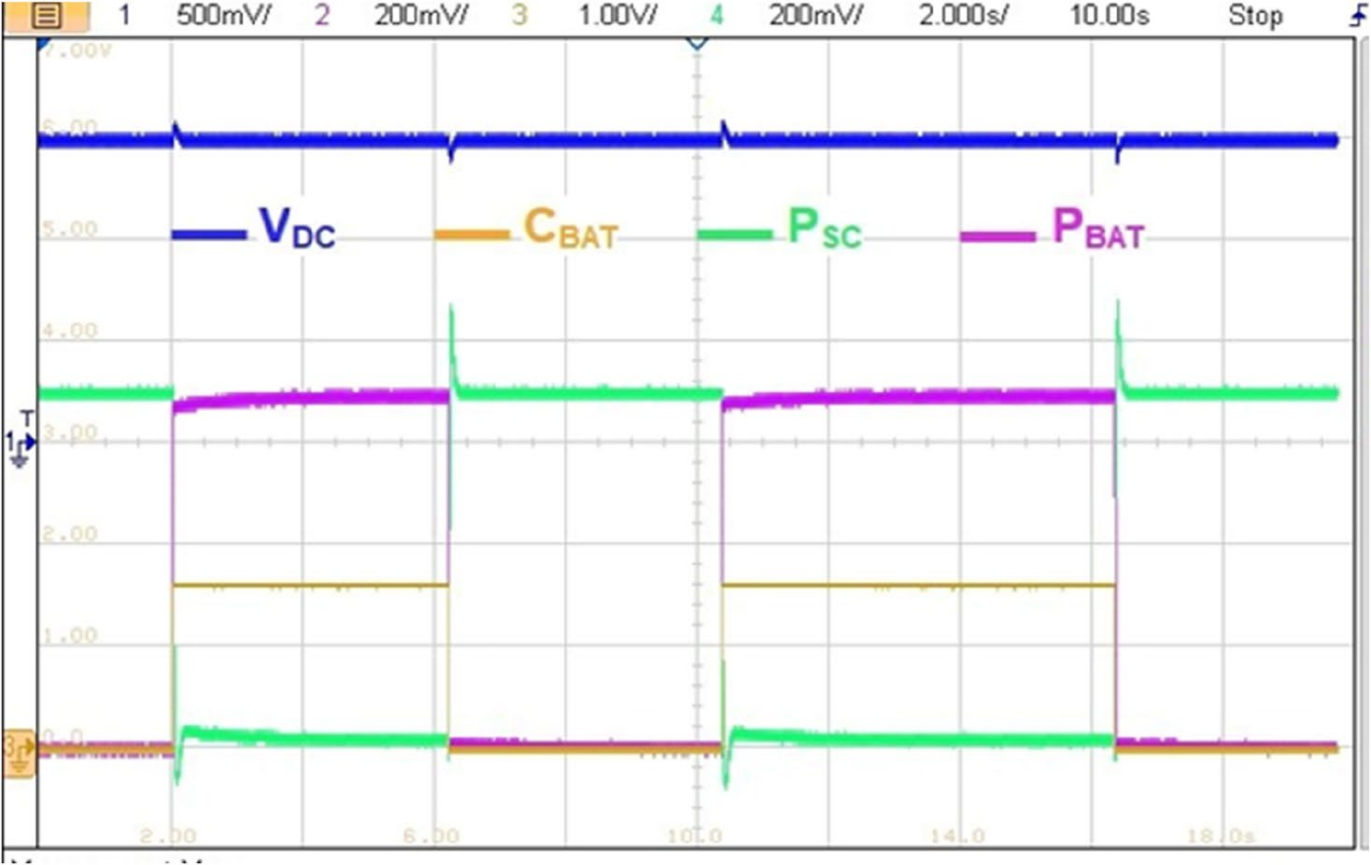
Effect of non-coordinated switching on DC bus voltage.

**Figure 34:**
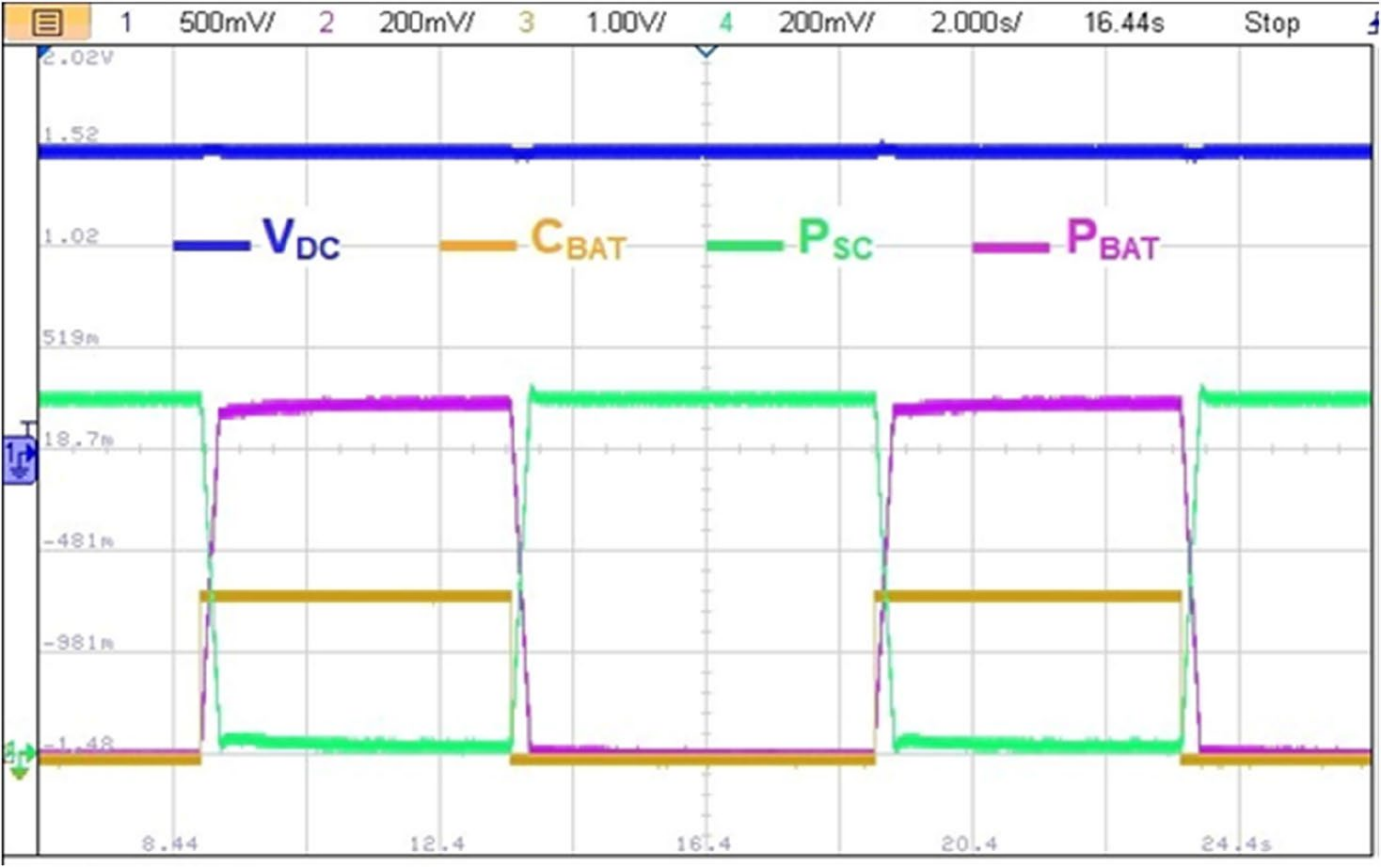
Effect of coordinated switching on DC bus voltage.

**Figure 35:**
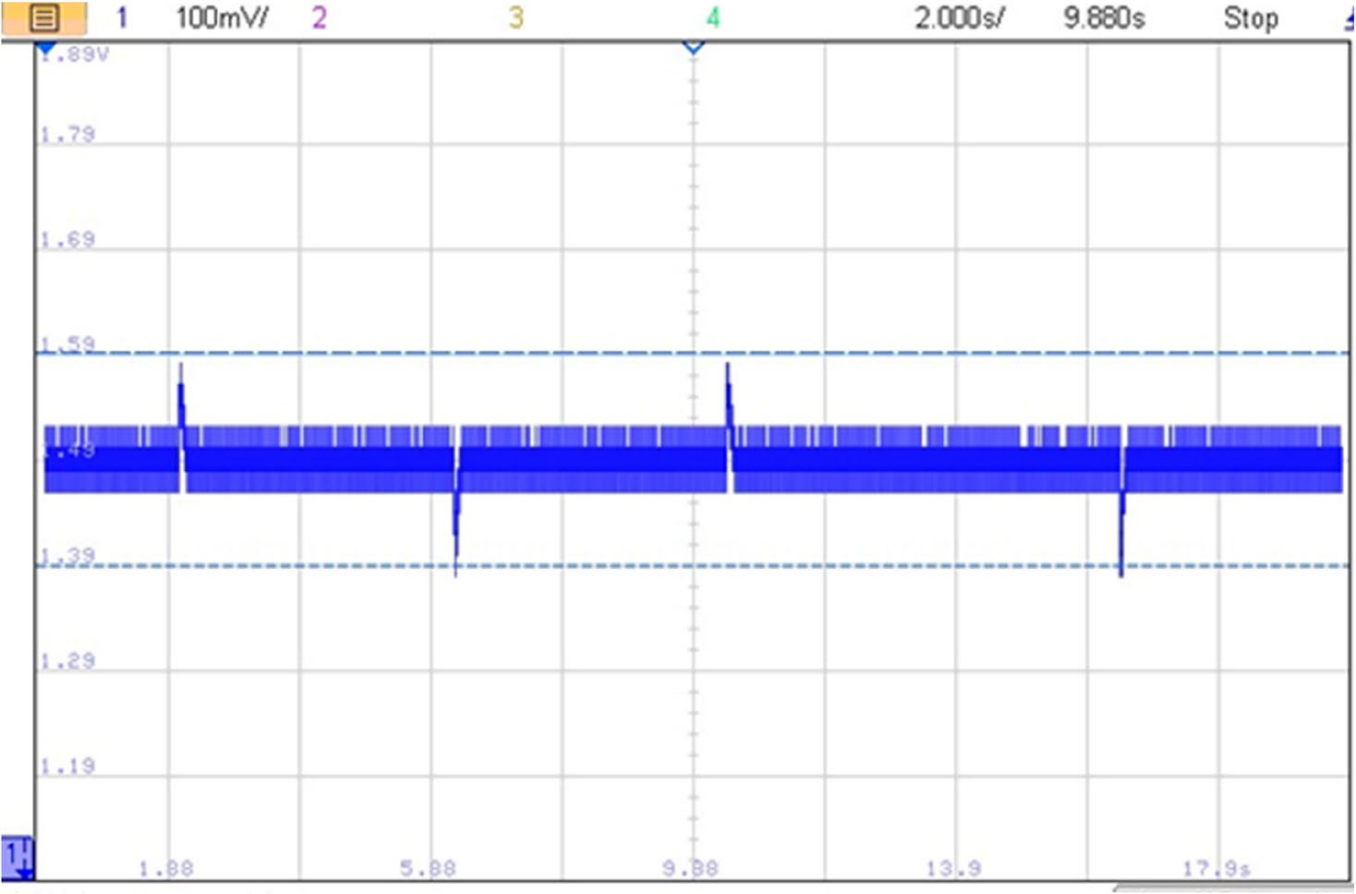
DC bus voltage zoom using abrupt switching.

**Figure 36:**
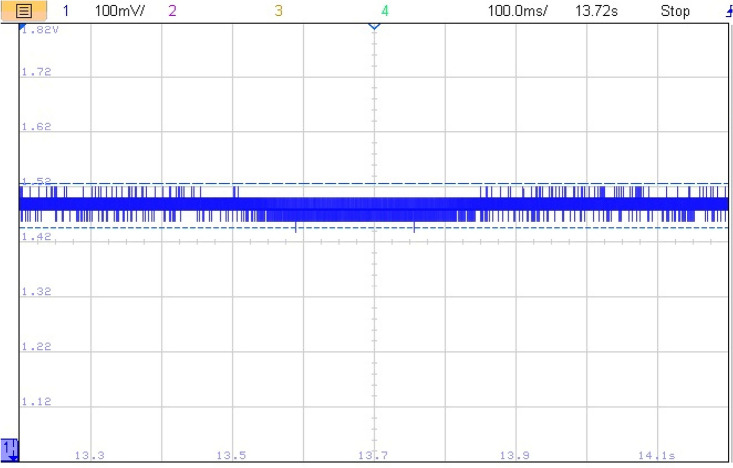
Effect of coordinated switching on DC bus voltage.

The impact of abrupt and coordinated switchings on DC bus voltage is shown in Figs. 33 and 34, respectively. Comparison between these two last mentioned figures shows that abrupt power source switching resulted in higher voltage fluctuations. As it can be seen in Fig. 33, the value of the DC voltage is equal to 1.5 V. The aforementioned value is obtained after multiplying the real DC bus voltage which is equal to 560 V times two scaling factors *G*_*1*_ and *G*_*2*_*. G1* is a scaling factor used to make the input voltage (Vin = 560 V) fall withing the tolerated RT LAB ranges (∓ 15 V). *G2* is the RT LAB calibration factor equal to 0.1. Abrupt switching resulted in a voltage ripple of 200 mV which corresponds to 74 V as it is shown in Fig. 35 whereas coordinated switching resulted in a ripple band of width less than or equal to 60 mV as it can be seen in Fig. 36. This proves that the proposed switching strategy minimizes DC bus fluctuations.

## Conclusion

The coordinated switching strategy outlined in this paper serves to elevate vehicle driving comfort by mitigating transient torque ripples and minimizing the undesirable passenger-perceived jerks during drive mode transitions. Furthermore, it allows a significant minimization of transient power ripples when switching between power sources with different transient dynamics such as batteries and supercapacitors. The coordinated switching strategy elucidated in this paper affords control over the duration of the switching period, providing designers the flexibility to select transition functions that align with the dynamics of power sources undergoing transitions. Thanks to the proposed coordinated switching strategy, harmful transient torque peaks were significantly reduced and power source transient DC bus ripples were reduced from 100 to 6 V and this is expected to enhance power sources lifespan. As a further work, it is intended to investigate the effectiveness of other transition function and to study the effect of on power sources and traction machine degradation.

## Data Availability

The datasets used and/or analysed during the current study available from the corresponding author on reasonable request.

## List of symbols

*F*_*ext*_: External force (N)
*m*: Vehicle mass
*a*: Acceleration (m/s^2^)
*F*_*T*_: Traction force (N)
*F*_*R*_: Resistive force (N)
*F*_*aero*_: Aerodynamic force (N)
*F*_*slope*_: Slope force (N)
*F*_*tire*_: Tire force (N)
*F*_*acc*_: Acceleration force (N)
*A*: Vehicle frontal area (m^2^)
*C*_*d*_: Aerodynamic drag coefficient (s^2^/m^2^)
*V*_*wheel*_: Vehicle longitudinal speed (m/s)
*V*_*wind*_: Wind speed (m/s)
*g*: Gravity acceleration (m/s^2^)
*f*_*r*_: Ground rolling resistance coefficient
*t*_*a*_: Time characteristic of the vehicle (s)
*T*_*LF*_: Load front torque (N m)
*T*_*LR*_: Load rear torque (N m)
*T*_*L*_: Total load torque (N m)
*r*: Wheel radius (m)
*G*: Gear ratio
*c*_*m1*_: PMSM1torque contribution factor
*c*_*m2*_: PMSM2 torque contribution factor
*T*_*HEV*_: Total developed electromagnetic torque (N m)
*T*_*m1*_: PMSM1 developed torque (N m)
*T*_*m2*_: PMSM2 developed torque (N m)
*T*_*S*_: Sampling time (s)
*T*_*n*_: Nominal torque (N m)
*t*_*trig*_: Triggering instant (s)
*T*_*SWM*_: Drive mode transition period (s)
*T*_*SWS*_: Power source transition period (s)
*T*_*TH*_: Torque threshold (N m)
*n*: Number of subtransient torque periods (s)
*m*: Number of subtransient power periods (s)
*K*_*i*_: *i*th Front positionner gear
*G*_*i*_: *i*th Rear positionner gear
$${L}_{d}$$: D-axis inductance (H)
$${L}_{q}$$: Q-axis inductance (H)
*i*_*q*_: Q-axis stator current (A)
*i*_*d*_: D-axis stator current (A)
*i*_*bat*_: Battery current (A)
*i*_*SC*_: Supercapacitor current (A)
*r*_*s*_: Stator resistance (Ω)
*w*_*r*_: Motor mechanical speed (rad/sec)
*p*: Number of pair of poles
*J*: Rotor inertia (Kg m^2^)
*B*: Mechanical damping coefficient (N m s)
*V*_*S*_: Stator voltage (V)
*V*_*dc*_: DC bus voltage (V)
*P*_*BAT*_: Fuel Cell power (W)
*P*_*SC*_: Supercapacitor power (W)
*P*_*HEV*_: Developed vehicle power (W)

## Greek letters

*ρ*: Air density (kg/m^3^)
*α*: Road inclination angle in (radian)
$\delta$: Drive mode subtransient period (s)
${\delta }_{FC}$: Fuel cell subtransient period (s)
${\delta }_{SC}$: Supercapacitor subtransient period (s)
*ξ*: Subtransition gear transition period
${\lambda }_{d}$: D-axis stator flux linkage (Wb)
${\lambda }_{q}$: Q-axis stator flux linkage (Wb)
$\phi$: Stator flux (Wb)
${\phi }_{PM}$: Flux due to permanent magnet (Wb)
${\phi }_{s\alpha }$: α-Axis stator flux (Wb)
${\phi }_{s\beta }$: β-Axis stator flux (Wb)
*θ*_*S*_: Stator flux angle (rad)

## Abbreviations

4WD: Four wheel drive
BAT: Battery
HEV: Hybrid electric vehicle
DDHEV: Distributed drive hybrid electric vehicle
DTC: Direct torque control
DC: Direct current
GHG: Greenhouse gas
IM: Induction machine
IGBT: Insulated bipolar gate transistor
PMSM: Permanent magnet synchronous machine
RES: Renewable energy sources
RWD: Rear wheel drive
SC: Supercapacitor
SOC_SC_: Supercapacitor state of charge
SOC_B_: Battery state of charge
P_F_: Front positioner
P_R_: Rear positioner
